# The FBXW7‐NOTCH interactome: A ubiquitin proteasomal system‐induced crosstalk modulating oncogenic transformation in human tissues

**DOI:** 10.1002/cnr2.1369

**Published:** 2021-04-06

**Authors:** Rohan Kar, Saurabh Kumar Jha, Shreesh Ojha, Ankur Sharma, Sunny Dholpuria, Venkata Sita Rama Raju, Parteek Prasher, Dinesh Kumar Chellappan, Gaurav Gupta, Sachin Kumar Singh, Keshav Raj Paudel, Philip M. Hansbro, Sandeep Kumar Singh, Janne Ruokolainen, Kavindra Kumar Kesari, Kamal Dua, Niraj Kumar Jha

**Affiliations:** ^1^ Indian Institute of Management Ahmedabad (IIMA) Ahmedabad Gujarat 380015 India; ^2^ Department of Biotechnology, School of Engineering & Technology (SET) Sharda University Greater Noida Uttar Pradesh 201310 India; ^3^ Department of Pharmacology and Therapeutics, College of Medicine and Health Sciences United Arab Emirates University Al Ain 17666 United Arab Emirates; ^4^ Department of Life sciences, School of Basic Science & Research (SBSR) Sharda University Greater Noida Uttar Pradesh 201310 India; ^5^ Department of Medical Biochemistry and Microbiology Uppsala University Uppsala Sweden; ^6^ Department of Chemistry University of Petroleum & Energy Studies Dehradun 248007 India; ^7^ Department of Life Sciences School of Pharmacy, International Medical University (IMU), Bukit Jalil Kuala Lumpur 57000 Malaysia; ^8^ School of Pharmacy Suresh Gyan Vihar University, Jagatpura Jaipur 302017 India; ^9^ School of Pharmaceutical Sciences Lovely Professional University Phagwara Punjab 144411 India; ^10^ Centre for Inflammation Centenary Institute New South Wales 2050 Australia; ^11^ School of Life Sciences, Faculty of Science University of Technology Sydney 2007 Australia; ^12^ Priority Research Centre for Healthy Lungs Hunter Medical Research Institute (HMRI), University of Newcastle, New Lambton Heights New South Wales 2308 Australia; ^13^ Indian Scientific Education and Technology Foundation Lucknow Uttar Pradesh 226002 India; ^14^ Department of Applied Physics, School of Science Aalto University Espoo Finland; ^15^ Discipline of Pharmacy, Graduate School of Health University of Technology Sydney NSW 2007 Australia

**Keywords:** cancer, diagnostic markers, E3 ligase, FBXW7, mutation, NOTCH, SCF, therapeutics

## Abstract

**Background:**

Ubiquitin ligases or E3 ligases are well programmed to regulate molecular interactions that operate at a post‐translational level. Skp, Cullin, F‐box containing complex (or SCF complex) is a multidomain E3 ligase known to mediate the degradation of a wide range of proteins through the proteasomal pathway. The three‐dimensional domain architecture of SCF family proteins suggests that it operates through a novel and adaptable “super‐enzymatic” process that might respond to targeted therapeutic modalities in cancer.

**Recent findings:**

Several F‐box containing proteins have been characterized either as tumor suppressors (FBXW8, FBXL3, FBXW8, FBXL3, FBXO1, FBXO4, and FBXO18) or as oncogenes (FBXO5, FBXO9, and SKP2). Besides, F‐box members like βTrcP1 and βTrcP2, the ones with context‐dependent functionality, have also been studied and reported. FBXW7 is a well‐studied F‐box protein and is a tumor suppressor. FBXW7 regulates the activity of a range of substrates, such as c‐Myc, cyclin E, mTOR, c‐Jun, NOTCH, myeloid cell leukemia sequence‐1 (MCL1), AURKA, NOTCH through the well‐known ubiquitin‐proteasome system (UPS)‐mediated degradation pathway. NOTCH signaling is a primitive pathway that plays a crucial role in maintaining normal tissue homeostasis. FBXW7 regulates NOTCH protein activity by controlling its half‐life, thereby maintaining optimum protein levels in tissue. However, aberrations in the FBXW7 or NOTCH expression levels can lead to poor prognosis and detrimental outcomes in patients. Therefore, the FBXW7‐NOTCH axis has been a subject of intense study and research over the years, especially around the interactome's role in driving cancer development and progression. Several studies have reported the effect of FBXW7 and NOTCH mutations on normal tissue behavior. The current review attempts to critically analyze these mutations prognostic value in a wide range of tumors. Furthermore, the review summarizes the recent findings pertaining to the FBXW7 and NOTCH interactome and its involvement in phosphorylation‐related events, cell cycle, proliferation, apoptosis, and metastasis.

**Conclusion:**

The review concludes by positioning FBXW7 as an effective diagnostic marker in tumors and by listing out recent advancements made in cancer therapeutics in identifying protocols targeting the FBXW7‐NOTCH aberrations in tumors.

## 1 BACKGROUND

**FIGURE 1:**
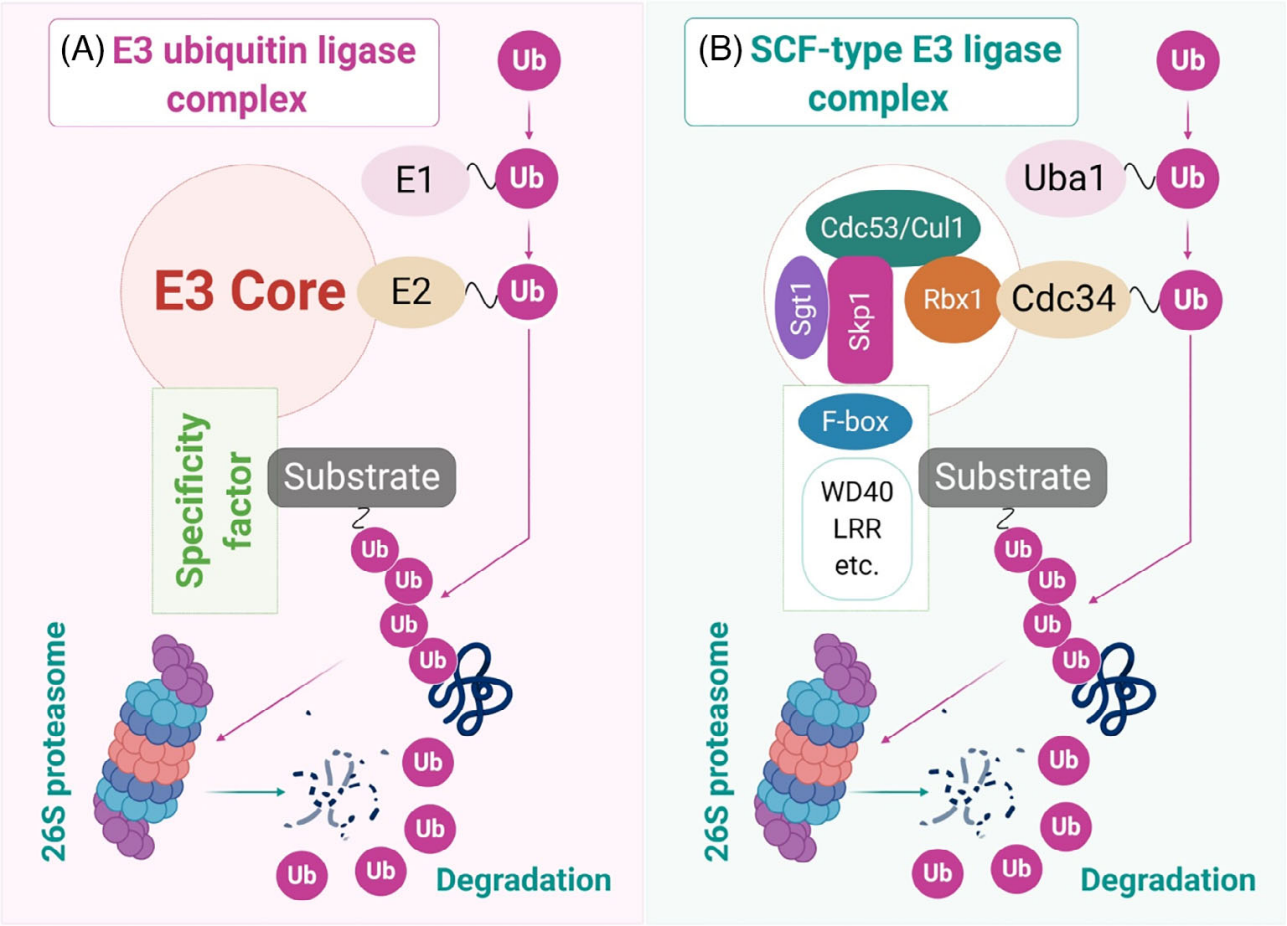
Domain architecture of E3 ubiquitin ligase and SCF‐type E3 ligase complex: A, common architecture underlies E3 ubiquitin ligase complex that mediate the targeted degradation of many cellular proteins. In targeting substrate proteins for degradation, ubiquitin is passed from an E1 ubiquitin‐activating enzyme to an E2 ubiquitin‐conjugating enzyme to the protein substrate, with the final step (ligating ubiquitin to the substrate) catalyzed by an E3 ubiquitin ligase. B, The SCF complexes are known to be E3 ligases and each complex in SCF‐type E3 ligase interacts with a set of adapter proteins that recruit different binding partners through specific protein‐protein interaction domains such as WD40 repeats and Leucine‐rich repeats (LRR) and facilitate substrates for proteasomal degradation. This figure was created by an author (N.K.J.) using the website https://app.biorender.com [Correction added on 27 April 2021, after first online publication: A typo within Figure 2 has been corrected]

The Skp, Cullin, F‐box containing complex (SCF) type multi‐subunit E3 ligase is the largest family of ubiquitin ligases, which facilitate the turnover of approximately 20% of all ubiquitin‐proteasomal system (UPS)‐regulated proteins in the mammalian system, including those critically involved in tumorigenesis. The SCF family of ubiquitin ligases (SCFCdc4) was first studied in budding yeast (*Saccharomyces cerevisiae*) by using the *in vitro* reconstitution technique. Thereafter, its complete structure was deciphered, and SCF was found to be a variable complex made up of S‐phase kinase‐associated protein 1 (Skp‐1), Cullins, F‐box proteins, and RBX/ROC RING finger proteins **(**Figure 1 **)**. Cullins act as a scaffold and comprises sequences that bind Skp‐1 and F‐box proteins at its N‐terminus and the RING proteins at its C‐terminus. The RING protein family consists of only two evolutionary conserved members, RBX1 (RING box protein 1), also known as ROC1 (regulator of Cullins), and RBX2/ROC2 (also known as SAG [sensitive to apoptosis gene]) that are necessary for driving the catalytic activity of SCF. F‐box proteins are the most critical element of the SCF assembly that is tasked with defining the substrate specificity of the SCF complex. The mammalian genome comprises 69 F‐box proteins that include the WD40 domain‐containing FBXWs, leucine‐rich repeats‐containing FBXLs and FBXOs (made up of diverse domains), and 7 Cullins (Cul‐1, 2, 3, 4A, 4B, 5, and 7). FBXW7 is the most widely studied F‐box protein due to its role in a panel of both normal and malignant cellular processes. FBXW7 binds to Skp1 and Cullins through its F‐box domain, and to the substrates through WD40 or leucine‐rich domains. Overall, Cullins‐RBX/ROC acts as the core of the SCF complex where RBX binds to ubiquitin‐conjugating enzymes (E2) and moderate the ubiquitin transfer from E2 to F‐box‐targeted substrates. The activity of SCF E3 ligases is also driven by events such as Cullin neddylation that disturbs the inhibitory binding of Cullin by Cullin‐associated NEDD8‐dissociated protein 1 (CAND1). Altogether, Cullin‐based SCF assembly can be divided into four categories, Cul1‐Skp1‐F‐box, Cul2/5‐Elongins‐B/C‐VHL/SOCS box, Cul3‐BTB, and Cul4A/B‐DDB1‐DWD, thereby making SCF the largest family of E3 ligases.^1^, ^2^, ^3^

FBXW7 (F‐box with seven tandem WD40; also known as Fbw7, Sel‐10, hCdc4, hAgo, or Archipelago), encoded by the gene *FBXW7* (loci, 4q31q.3), was first isolated as Cdc4 in budding yeast. The gene comprises 4 untranslated regions (UTRs) and 13 coding exons, which altogether define the substrate specificity of FBXW7, primarily through alternate splicing mechanisms. The human *FBXW7* gene is reportedly deleted in approximately 30% of all cancer types, thereby underscoring its role in tumor progression and prognosis. Mammalian cells express three FBXW7 isoforms, FBXW7α, FBXW7β, and FBXW7γ, and each of these isoforms is localized in a tissue‐specific manner to restrict their interaction to specific substrates. Broadly, FBXW7α is found in abundance in the nucleoplasm, as compared to FBXW7β, which is cytoplasmic and FBXW7γ that is nucleolar. FBXW7α is the most abundant isoform that facilitates the degradation of most FBXW7‐associated substrates. Furthermore, FBXW7α is ubiquitously expressed in a range of proliferating cells and tumor types and is capable of performing almost all the known functions of FBXW7. On the other hand, FBXW7β helps in protecting the cells from damaging events like oxidative stress (OS). These isoforms can be structurally distinguished based upon their N‐terminal region (NTR) sequences and shared conserved domain sequences in their C‐terminal region (CTR). Nevertheless, all of these FBXW7 isoforms comprise three essential domains, the dimerization domain, the F‐box domain, and 7‐8 tandem WD40 repeats. The F‐box domain helps in binding FBXW7 to the Skp1 component of the SCF complex, and together, the dimerization and WD40 repeat domain (harboring three arginine residues); facilitate the FBXW7 binding to phosphorylated substrates for SCF‐mediated proteasomal degradation. Primarily, substrate recognition happens through recognition of conserved phosphorylated glycogen synthase kinase 3 (GSK3)‐mediated domain called the Cdc4 phosphodegrons (CPDs).^4^, ^5^ Activated GSK3 phosphorylates the CPDs present in target substrates, which subsequently bind to the WD40 repeats of FBXW7. GSK3‐mediated phosphorylation of CPD is an essential step in the interaction between the substrate and FBXW7, although the role of other kinases in the process is now being explored. Mutations within the CPD motif have been observed in several cancers that lead to the stabilization of oncogenic substrates, like c‐Myc and NOTCH. Sequence analysis of FBXW7 substrates has identified the conserved CDP motif to be ΦXΦΦΦ‐T/S‐PPX‐S/T/E, where Φ is a hydrophobic residue and X could be any amino acid. It must be noted that the phosphorylation of S/T/E residue triggers the GSK3‐mediated phosphorylation of T/S residue. Some reports suggest the existence of more than one CPD motif within FBXW7 substrates, like Cyclin E that comprise two CPD motifs, one located in the T380 position, and the other being centered around ~T62. Together, these two CPD motifs improve the efficiency of FBXW7 binding with Cyclin E in response to various signaling cues.^6^ Some studies highlight the antagonistic behavior of FBXW7α and FBXW7β toward substrates. For instance, peroxisome proliferator‐activated receptor‐gamma co‐activator (PGC)‐1α, a transcriptional co‐activator with widespread effects on cellular energy metabolism undergoes different fate under FBXW7α and FBXW7β, respectively. Cellular PGC‐1α is downregulated by FBXW7β while FBXW7α upregulates it through a ubiquitin‐mediated stabilization.^7^, ^8^, ^9^ Overall, the biology of FBXW7 is very complex not only in normal cells but in a wide range of carcinomas as well. It is therefore essential to understand the FBXW7 based interactions with much more clarity to identify disease‐critical interactions, which potentially drive pathogenesis in cancer patients.

**FIGURE 2:**
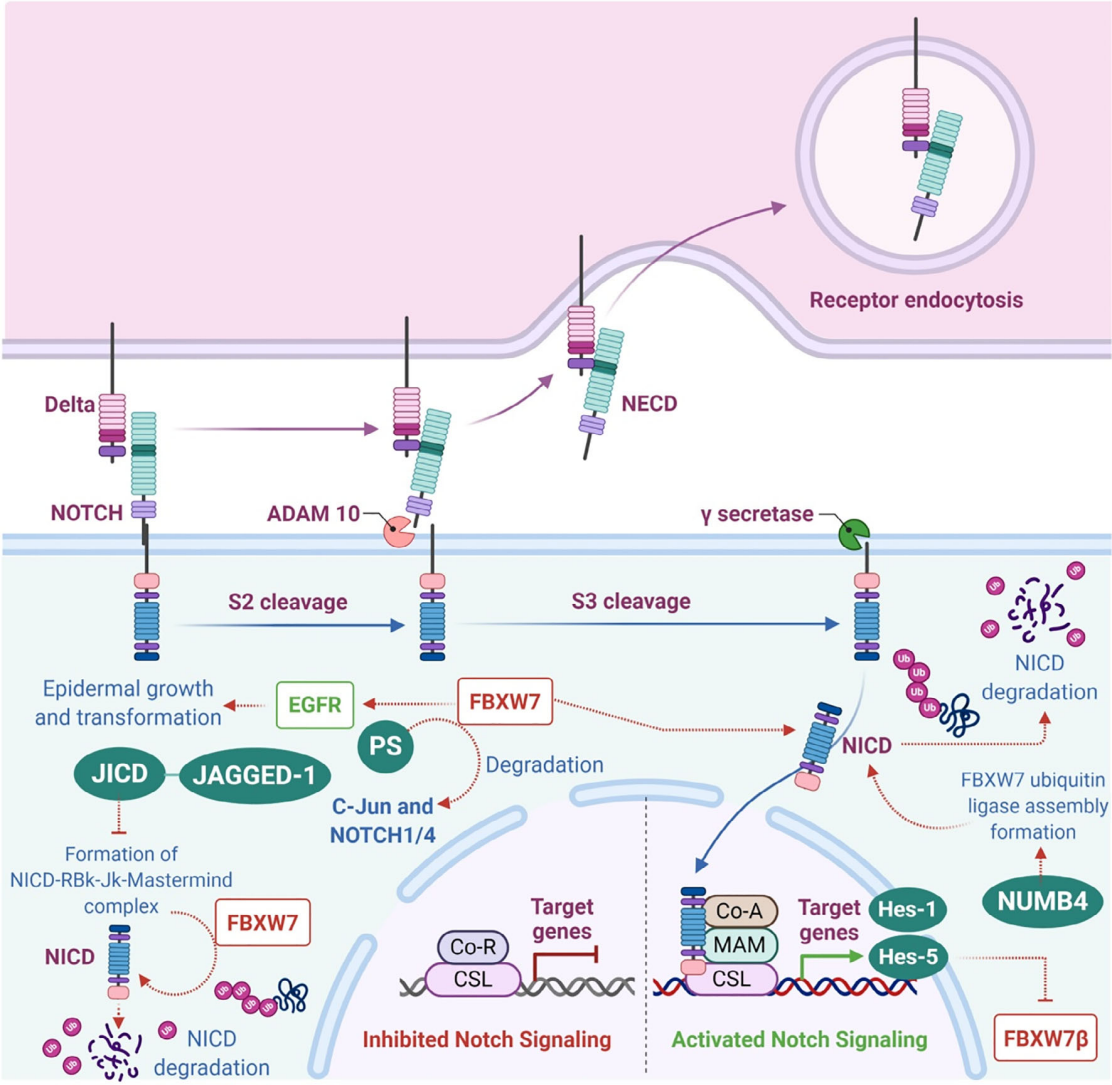
NOTCH Pathway and FBXW7‐dependant degradation of NOTCH intracellular domain (NICD). NECD, Notch extracellular domain; EGFR, epidermal growth factor receptor; JICD, JAGGED1 intracellular domain; CSL, CBF1, Suppressor of Hairless, Lag‐1; Co‐R, co‐repressor; Co‐A, co‐activator; MAM, Mastermind‐like (MAML) transcriptional co‐activators. This figure was created by an author (N.K.J.) using the website https://app.biorender.com [Correction added on 27 April 2021, after first online publication: A typo within Figure 2 has been corrected]

The effect of E3 ligases on the NOTCH pathway is well known. Members of several E3 ligase families interact either directly or indirectly with NOTCH and with other NOTCH pathway‐associated proteins to facilitate a panel of cellular processes. Therefore, subtle deregulation in the UPS, including the E3 ligases, coupled with NOTCH‐based aberrations can disturb normal cellular homeostasis and may drive the cell to a malignant fate. For example, conditional inactivation of Mind bomb‐1 (Mib1), a ubiquitin ligase mostly associated with the endocytosis of Delta receptor in the mouse brain, results in the complete loss of NOTCH activity, thereby hampering neural stem cell differentiation. Likewise, Drosophila Su(dx), a member of the homologous to E6‐associated protein C terminus (HECT) family of ubiquitin ligases is known to negatively regulate the NOTCH pathway. Even further, the mammalian ortholog of Su(dx), Itch (atrophin‐interacting protein 4), has been known to facilitate the polyubiquitination of the membrane‐anchored form of NOTCH, leading to its lysosomal degradation.^10^, ^11^, ^12^ Several studies have confirmed that the NOTCH intracellular domain (NICD) is the principal target of degradation by the SCF family E3 ligases FBXW7 **(**Figure 2 **).** An initial study on Sel‐10 (nematode homolog of FBXW7) found that NICD is negatively regulated by FBXW7, which results in deactivation of the NOTCH pathway. Over the last decade or so, NOTCH and FBXW7 mutations have been reported in a wide range of tumors, such as pediatric and adult T‐cell acute leukemia (T‐ALL), diffuse large B‐cell lymphoma (DLBCL), splenic marginal zone lymphoma (SMZL), Hajdu‐Cheney syndrome, breast cancer (BC), non‐small‐cell lung carcinomas (NSCLCs), etc. Given the array of pathological events, which may be driven by a deregulated FBXW7‐NOTCH interactome in tumors, it is imperative to understand such disease‐critical interactions with a therapeutic perspective in mind.^13^

## 2 KEY INTERACTIONS BETWEEN FBXW7 AND NOTCH PATHWAY ASSOCIATED COMPONENTS

Although NICD remains the principal substrate of FBXW7 with respect to the NOTCH pathway, there are emerging evidences that highlight the interaction between FBXW7 and other accessory proteins that are directly or indirectly involved in various key cellular processes and in the discharge of NOTCH signaling duties (Table 1 and Figure 2). This section presents an insight into such novel interactions involving FBXW7 and proteins, such as Presenilins (PS), Hes‐1, Hes‐5, etc.

**TABLE 1:** List of interacting components of FBXW7 and NOTCH and their associated functions in various key cellular processes

Key processes	Interacting components	Functions	References
Keratinocyte development and Skin tumorigenesis	FBXW7, NOTCH, and c‐Myc	FBXW7 regulates the proliferation and differentiation of keratinocytes, and exerts both inhibitory and stimulatory signals during skin carcinogenesis, primarily by counteracting the proliferation‐advancing activity of c‐Myc and the tumor‐suppressive activity of NOTCH.	^14^
Intestinal homeostasis	FBXW7, NOTCH, Jun, and DEK	FBXW7 mutation leads to accumulation of multiple substrates and results in improper degradation of NOTCH, Jun, and DEK, which can contribute to intestinal carcinogenesis.	^15^
Oligodendrocyte development	FBXW7 and NOTCH	FBXW7 helps minimize NOTCH activation during zebrafish neural development, thereby limiting the number of oligodendrocyte progenitor cells (OPCs).	^16^
Melanoma	FBXW7 and NOTCH	FBXW7 as a critical tumor suppressor that is reportedly mutated and inactivated in melanoma, resulting in constitutive NOTCH1 activation.	^17^
Mouse embryonic fibroblasts (MEFs) development	FBXW7 and NOTCH	FBXW7 does not operate as an onco‐suppressor in MEFs. Rather, it facilitates cell cycle progression and cell survival through degradation of NOTCH1 by a FBXW7‐dependant mechanism.	^18^
Angiogenesis	FBXW7 and NOTCH	FBXW7 is a positive regulator of angiogenesis that restricts the activity of NOTCH in the endothelium of the growing vasculature.	^19^
Lipid Metabolism	FBXW7 and RBP‐J	FBXW7 plays key roles along with NOTCH cofactor RBP‐J in modulating lipogenesis, cell proliferation and differentiation in the liver.	^20^
Bipotent liver progenitor cells (LPCs) differentiation	FBXW7, NOTCH, and Hdac1	Hdac1 regulates the differentiation of LPCs into hepatocytes via Sox9b, and into BECs through Cdk8, FBXW7, and NOTCH3 in zebrafish models.	^21^
Neural cell differentiation	FBXW7, NOTCH, and c‐Jun	FBXW7 regulates neurogenesis by antagonizing NOTCH and c‐Jun N‐terminal kinase (JNK)/c‐Jun pathway.	^22^
Osteoporosis	FBXW7 and NOTCH	Overexpression of NOTCH2 carrying a truncated C terminus that escapes FBXW7‐mediated degradation results in sustained osteoclast activity.	^23^
Neural stem cell fate determination	FBXW7, NOTCH, and Hes‐5	FBXW7β transcription by NOTCH signaling provides an essential mechanism that is coupled to and absolutely essential for the correct specification of cell fates triggered by lateral inhibition.	^24^
Cell fate, proliferation, and homeostasis	FBXW7, NOTCH, and Alpha‐synuclein (SNCA)	SNCA promotes degradation of NOTCH1 intracellular domain (NICD) by FBXW7‐dependant mechanism, and attenuates the interaction between NICD and RBP‐Jk.	^25^
EGFR signaling and cell transformation	FBXW7, NOTCH, and PS	PS plays a key role during epidermal growth and transformation by reciprocally modulating the EGFR and NOTCH signaling through FBXW7.	^26^

### 2.1 Presenilins

Presenilins (PS) are the catalytic components of the enzyme γ‐secretase that is needed for NOTCH pathway activation. It has been suggested that FBXW7α positively regulates the activity of NICD and epidermal growth factor receptor (EGFR), principally by striking a balance between ubiquitylation and stability of both NICD and EGFR. PS also negatively regulates FBXW7 transcriptional activity, thereby positively and negatively controlling EGFR and NOTCH pathway, respectively. Researchers utilizing a novel epidermal conditional PS‐deficient mice (PS deactivation in keratinocytes of the basal layer of the epidermis) reported epidermal hyperplasia in rodents along with upregulation of EGFR and FBXW7 expression and downregulation of NICD in keratinocytes. These observations emphasize on the fact that FBXW7 can act as a central mediator of the upstream PS and the downstream EGFR and NOTCH pathways.^27^Incidentally, EGFR and NOTCH signaling pathways have opposing roles during epidermal differentiation and tumorigenesis and PS plays a fundamental role in the process. Loss of PS activity results in enhanced EGFR‐dependent signaling and cellular proliferation that drives oncogenic transformation in mice, which can be directly attributed to the γ‐secretase‐independent transcriptional upregulation of FBXW7. Moreover, FBXW7α that tags NICD for degradation also can facilitate the positive EGFR activity by controlling the ubiquitination step necessary for constitutive degradation and stability of EGFR. Furthermore, loss of PS function in fibroblasts and primary keratinocytes results in FBXW7 overexpression and subsequent degradation of its substrates, such as c‐Jun and NOTCH1/4. Collectively, these findings propose a novel role of PS during epidermal growth and transformation, essentially involving an FBXW7‐mediated reciprocal regulation of the EGFR and NOTCH signaling cascades. However, it must be noted that the PS‐dependent regulation of FBXW7 and EGFR is totally independent of its proteolytic activity as inhibition of γ‐secretase has been found to have no effect on the expression and activity of both FBXW7 and EGFR. Interestingly, these findings differ from the well‐established hypothesis that FBXW7 by default acts as a tumor suppressor in several cancers including epithelial tumors. This recent study is the first of its kind to propose a crosstalk involving PS, EGFR, NOTCH, and FBXW7 in skin cancers.^26^

### 2.2 Hes‐1 and Hes‐5

Overexpression of NOTCH target genes has been reported in a range of human cancers. Hes‐1 and Hes‐5 are known downstream effectors of NOTCH signaling and their expression levels are highly upregulated in a range of cancer types. Due to their deregulated expression patterns, both Hes‐1 and Hes‐5 have been explored as potential diagnostic markers in patients with advanced‐stage tumors. A recent study by Sancho et al. found that Hes‐5 directly represses the transcriptional activation of FBXW7β. Notably, the study highlighted the existence of a FBXW7β/NICD/Hes‐5 positive feedback loop that underlies FBXW7 haploinsufficiency in tumors.^28^ The existence of a similar feedback regulatory loop was confirmed by another research group that reported that FBXW7(Δ/+) heterozygous mice display haploinsufficiency for NOTCH degradation that invokes intestinal progenitor cell and neural stem cell differentiation. The potential involvement of Hes‐5 was attested by the fact that concomitant inhibition of Hes‐5 improved phenotypes and restores the order of normal stem cell differentiation. FBXW7 itself is transcriptionally downregulated by the NOTCH downstream effector Hes‐5, and therefore, the perturbation of the feedback regulatory loop may be held responsible for the FBXW7 haploinsufficiency as seen for NOTCH‐dependent functions in the cancer stem cells (CSCs). Collectively, these observations reveal the non‐unidirectional nature of the functional relationship between FBXW7β and NICD, and the potential existence of a double‐negative, that is, positive feedback loop involving Hes‐5. However, it remains to be seen whether Hes‐1 participates in the feedback loop, and in what capacity.^24^

### 2.3 NUMB

NUMB was originally reported to be essential for cell fate determination during the neuroblast division. However, very recently, NUMB was designated as a bonafide tumor suppressor gene in several human carcinomas. For instance, in breast tumors, frequent loss of NUMB activity has been observed that can be related to poor prognosis. Likewise, NUMB activity has also been found to be downregulated in NSCLCs. Moreover, NUMB deletions and low NUMB expression levels have also been reported in pro‐neural glioblastomas. NUMB protein regulates a panel of signaling pathways, such as p53, NOTCH, and Hedgehog, and hence plays a key role during tumorigenesis. Studies have suggested that NUMB can bind to and represses the E3 ubiquitin ligase Mdm2, which is responsible for p53 degradation, consequently leading to an upregulation in cellular p53 levels. Moreover, NUMB can bind to the E3 ligase Itch that leads to the ubiquitination of NOTCH, suggesting that NUMB may act as a connecting link between NOTCH and Itch. It must be noted that NUMB itself serves as a substrate for several E3 ligases, like seven in absentia homolog 1 (Siah‐1) and ligand of Numb protein‐X (LNX). Although little is known about its interaction with FBXW7, a recent study found that one of the predominant NUMB isoform, NUMB4, helps in FBXW7 ubiquitin ligase assembly formation and activation, thereby resulting in enhanced NICD degradation and subsequent deactivation of the NOTCH pathway. Nevertheless, the precise mechanism behind such NUMB‐based interaction is still unknown and a further in‐depth investigation is required to understand the pathological contribution of NUMB4‐mediated control of FBXW7 and NOTCH signaling during carcinogenesis.^28^

### 2.4 JAGGED‐1

Not enough data is available to link the NOTCH pathway associated ligand JAGGED1 with FBXW7. After the initial ligand‐receptor interaction; NOTCH signaling is activated by a proteolytic cleavage of the NOTCH receptor. JAGGED1 undergoes a similar γ‐secretase‐mediated proteolytic cleavage that releases an intracellular fragment, JAGGED1 intracellular domain (JICD). JICD can block NOTCH1 signaling activation by decreasing the stability of the NICD. Moreover, the presence of JICD prevents the formation of the NICD‐RBP‐Jk‐Mastermind complex and also expedites the proteasomal degradation of NICD through the FBXW7‐dependent pathway. Altogether, these early findings suggest that JICD can operate as a negative regulator of NOTCH signaling by promoting the FBXW7‐mediated degradation of NICD. However, the precise mechanism behind such degradation and the exact role of JICD and other NOTCH ligands in facilitating the process remains an open area for further research.^29^

## 3 MUTATIONAL EVENTS DRIVING NOTCH AND/OR FBXW7 DEREGULATION IN DIFFERENT CANCER TYPES

**FIGURE 3:**
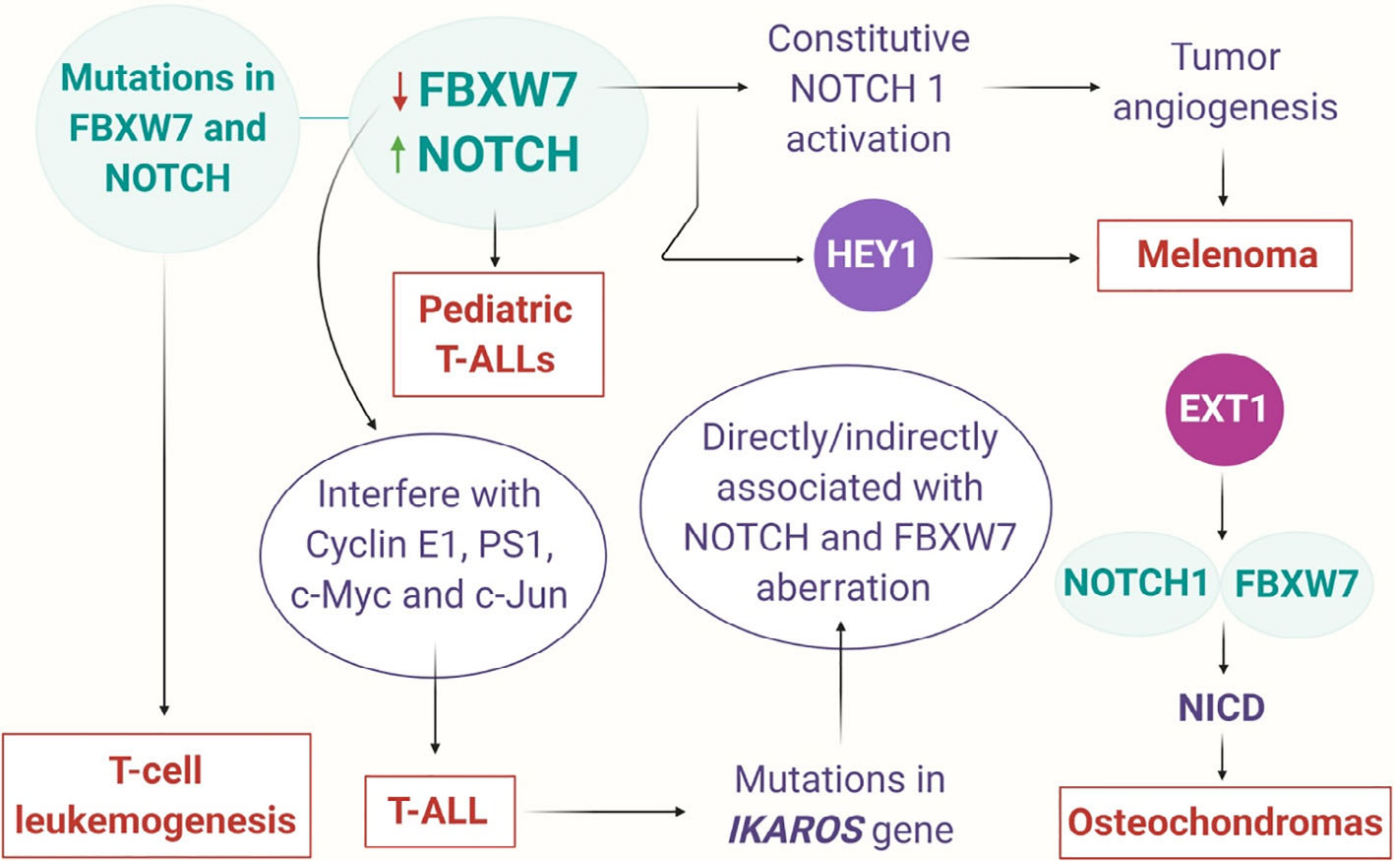
NOTCH signaling and FBXW7 deregulation in different cancer types. This figure was created by an author (N.K.J.) using the website https://app.biorender.com [Correction added on 27 April 2021, after first online publication: A typo within Figure 3 has been corrected]

Perturbed genotypes in cancer can now be studied using whole‐genome sequencing of diverse tumor tissues and the observed gene mutations can be further used for prognosis and classification of cancer subtypes. Although mutations in few disease‐drivers can be directly associated with key signaling pathway aberrations, a more comprehensive understanding of how proto‐oncogenes and tumor suppressors drive oncogenesis in humans is much more difficult to assess. Mutations have been reported widely in both NOTCH and FBXW7, and have been associated with aberrant activation of the NOTCH pathway and associated sequelae (Figure 3). The general observation is that NOTCH mutations result in the constitutive activation of the pathway, while FBXW7 mutations impair its normal functionality and results in the abnormal degradation of substrates, including NOTCH, c‐Myc, and c‐Jun. However, it is essential to decipher the precise mechanism by which these mutations can drive prognosis in patients. In the underlying sections, we present an extensive analysis of all these observed mutations including their prognostic relevance in different cancer forms.

### 3.1 T‐cell acute lymphoblastic leukemia

Precursor T‐cell acute lymphoblastic leukemia (T‐ALL) continues to remain a roadblock in pediatric oncology. T‐ALL tumor relapses display extremely poor prognosis, and therefore, it is vital to isolate molecular risk factors, which allow early and efficient treatment stratification. Activating mutations in the NOTCH gene indicates a poor prognosis in T‐ALL patients who can be attributed in part to the inactivation of FBXW7, which possibly synergize with NOTCH1 receptor activation. But, in some T‐ALL cases, the pathological effect of FBXW7 inactivation has been found to be separable from NOTCH1 activation, by not synergizing with activating NOTCH1 mutations, while presenting a more favorable long‐term outcome. NOTCH1 mutations have been reported in approximately 50% of human T‐ALLs, and missense mutations in FBXW7 have been reported in approximately 15% of T‐ALL cases. Mostly mutations in FBXW7 are localized in the evolutionary conserved side of the gene, and both the location and conservation statuses indicate that the novel missense mutations may affect FBXW7 substrate targeting. Furthermore, it has been reported that T‐ALL patients, expressing NOTCH1/FBXW7 double mutations, display a much favorable prognosis when compared to patients with mutations in either NOTCH1 or FBXW7. Nevertheless, the precise mechanism behind the separability of NOTCH1 receptor activation and FBXW7 inactivation remains an open question at this point. But, to an extent, this can be explained by the fact that activation of the NOTCH1 receptor is less pleiotropic than the inactivation of its negative regulator FBXW7, because FBXW7 not only interferes with the NOTCH pathway but also with a panel of other oncogenic pathways and proteins, such as Cyclin E1, PS1, c‐Myc, c‐Jun, Aurora‐A, sterol regulatory element‐binding protein (SREBP), etc.^30^, ^31^ The majority of NOTCH mutations observed in T‐ALLs belong to two major classes: point mutations that destabilize the NOTCH1 heterodimerization (HD) domain, thereby enhancing its cleavage by γ‐secretase; and disruption/deletion mutations of the C‐terminal PEST (proline, glutamic acid, serine, and threonine) domain, which drive NICD stabilization, thereby leading to the constitutive activation of the NOTCH1 cascade. It has been reported that when mutations are located at both HD and PEST sites, they are essentially in the cis loci of the NOTCH1 allele. FBXW7 mutations can be attributed to NOTCH1 HD mutations, suggesting that NOTCH pathway activation is one of their major roles in T‐cell leukemogenesis. Interestingly, FBXW7 mutations are rarely detected alongside a PEST mutation and it can be conferred that both FBXW7 and PEST mutations increase the stability of the N1‐ICD and therefore, may be functionally redundant.^32^, ^33^

Increased NOTCH or decreased FBXW7 expression can be associated with a favorable outcome in pediatric T‐ALLs. Recently, both the frequency and prognostic manifestation of mutations in NOTCH1 and FBXW7 were investigated in patients of Swedish childhood T‐ALL treated according to the Nordic Society of Pediatric Hematology and Oncology (NOPHO) ALL‐1992 and ALL‐2000 protocols. In a subgroup of patients, the functional relevance of NOTCH1 mutations was measured as a function of Hes‐1, c‐Myb, and c‐Myc expression. It was found that approximately 59% of the studied cases expressed mutations in NOTCH1 and/or FBXW7. Incidentally, no such difference was observed in overall or event‐free survival (EFS) in patients with T‐ALL expressing mutations in NOTCH or FBXW7 when compared to those without any mutations. Moreover, T‐ALL cells carrying NOTCH1 mutations displayed high Hes‐1 and c‐Myb transcriptional activity when compared to cells expressing wild‐type NOTCH1, thereby suggesting a hyperactivation of the NOTCH pathway. However, high Hes‐1 expression improved overall EFS in some cases, for which the mechanism remains to be studied further.^34^ Another study group extended the previously established Childhood Acute Lymphoblastic Leukemia (CoALL) protocol to a wider cohort and additionally included the Argentine T‐ALL patients from ALL IC‐BFM (Berlin‐Frankfurt‐Münster) protocol to potentially study novel mutations. The study revealed a stronger discriminatory effect on the genotype/phenotype relationship concerning early treatment response and long‐term outcome. Overall, it was found that approximately 60% of T‐ALL samples expressed at least one NOTCH1 mutation, and a few of them displayed both NOTCH1 and FBXW7 mutations. A very small fraction of the cohort displayed only FBXW7 mutations, while close to 30% of the cohort presented a wild‐type configuration of either gene. Interestingly, four new NOTCH1 mutations were identified in the C‐terminal PEST domain, in the rarely affected Lin12‐NOTCH (LNR) domain, and in the ankyrin repeat domain. These novel LNR mutations may provide a more comprehensive insight into the structure of the NOTCH1 negative regulatory region (NRR) and the R1946 mutation, identified in the ankyrin domain may serve as an unusual loss‐of‐function mutation. Furthermore, the more common HD and PEST domain mutations might individually exert distinct functional effects on the cellular homeostasis with prognostic implications.^35^ HD domain mutations drive subunit dissociation or trigger NOTCH1 to become sensitive to ligand‐independent cleavage, thereby resulting in increased NICD stabilization and translocation to the nucleus. HD mutations like L1575P limit the efficiency of furin‐mediated cleavage and induce the γ‐secretase‐dependent NOTCH1 activation. HD domain mutations are known to drive a substantial change in secondary structure of NOTCH by a non‐conservative amino acid substitution. Although this region is known to harbor most of the activating NOTCH mutations, it cannot be ignored that some of these may actually represent polymorphism. The PEST domain, which is located in the C‐terminal part of NOTCH1, regulates protein turnover and deletions in the PEST region can increase the stability of the protein, which results in upregulation of the NOTCH1 cascade. R465C and R479Q mutations in the conserved protein‐protein interaction domains of FBXW7 abrogate NOTCH1 binding that can lead to constitutive activation of NOTCH1. Interestingly, FBXW7 mutations have been mostly linked with the HD domain that causes signal amplification, whereas FBXW7 mutations in PEST region sequences are less common. Till date, there is no concrete evidence to suggest the presence of mutations both in the PEST domain and in the FBXW7 gene. Nevertheless, some research groups argue that mutations in the PEST domain could be a means to relieve mutational pressure on the FBXW7 gene.^31^ Study conducted on patients with adult T‐ALL treated on the United Kingdom Acute Lymphoblastic Leukaemia XII (UKALLXII)/Eastern Cooperative Oncology Group (ECOG), E2993 protocol found a notable positive correlation between a NOTCH1 mutation in the HD domain only and an FBXW7 mutation, and a strong negative correlation between NOTCH1‐PEST mutation and FBXW7 mutation. These data are consistent with an earlier study that reported that NOTCH1‐HD and FBXW7 mutations act in unison, similar to dual HD and PEST mutations. In spite of the fact that FBXW7 also targets c‐Myc for degradation, the relationship observed in this current study highlight the fact that FBXW7 mutations are acquired by T‐ALL cells to improve NOTCH1 signal strength. It must be noted that if FBXW7 mutations were expressed by tumor cells predominantly to upregulate c‐Myc activity and therefore, they are likely be detected in conjunction with PEST mutations, at least in some cases. Quite astonishingly, the current study found that NOTCH1/FBXW7 MUT and WT groups were equally balanced in terms of percentages of standard‐ and high‐risk patients, thereby implying a more discrete role of mutations in driving prognosis of T‐ALL.^36^

Lastly, in a few T‐ALL cases, mutations have been reported in the IKAROS gene, which can be associated directly or indirectly to NOTCH and/or FBXW7 aberrations. In mouse, T‐ALL‐activating NOTCH1 mutations usually collide with loss‐of‐function mutations in Ikzf1 gene that encodes the zinc‐finger transcription factor IKAROS that regulate hematopoietic stem cell functions and drive lineage fate decisions of early hematopoietic progenitors. Therefore, IKAROS dominant‐negative mice lack B‐cells, natural killer (NK) cells, and fetal T cells, and postnatally synthesize aberrant, clonally expanded T cells. Moreover, aggressive T‐cell carcinomas are observed in mice carrying the dominant‐negative or hypomorphic IKAROS alleles, suggesting a decisive role of the protein in T‐lineage tumor suppression. Incidentally, approximately 60 to 70% of all T‐ALLs originating in IKAROS germline mutant mice models display NOTCH1 mutations, such as PEST and/or HD domain mutations found in human T‐ALL. Beverly and Capobianco first proposed that IKAROS directly antagonizes NOTCH1 target gene activation, and also found that retroviral expression of the full‐length IKAROS isoform, Ik1, leads to cell cycle arrest that can be attributed to the downregulation of the canonical NOTCH target gene, Hes‐1. IKAROS binds to the Hes‐1 promoter and competes with RBPJ at Hes‐1 promoter sequences to repress NOTCH1‐mediated reporter gene expression. While these observations do indicate that IKAROS suppresses the NOTCH1 activation and subsequent transcription of downstream target genes like Hes‐1 in thymocytes, the precise role of FBXW7 mutations in these IKAROS‐mediated processes needs to be investigated further in T‐ALL tumors.^27^, ^37^

### 3.2 Adult T‐cell leukemia

Adult T‐cell leukemia (ATL) is an aggressive lymphoproliferative disease with an extremely poor prognosis. Several study groups have earlier reported the presence of NOTCH1 activating mutations and constitutive activation of the pathway in patients suffering from acute ATL but less is known about the FBXW7 mutations and their effect in ATL. Yeh et al. reported high frequency of FBXW7/hCDC4 mutations within the WD40 substrate‐binding domain in ATL cells. FBXW7 is a well‐known tumor suppressor that limits tumor cell proliferation, thereby supporting the absolute necessity for its inactivation in progressive tumors. Two FBXW7 mutants, D510E and D527G were found in ATL tumors, which retained their unique ability to target normal FBXW7 substrates, such as endogenous Cyclin E, MCL‐1, and c‐Myc. However, these mutants lost their ability to facilitate degradation of N1‐ICD in these ATL cell lines. These observations are in stark contrast with earlier studies where FBXW7 mutants have been found to abolish the degradation of all its substrates, including N1‐ICD. Further investigation revealed that D510E mutant retains the ability to interact with N1‐ICD but is ineffective in facilitating the K48‐mediated ubiquitination of N1‐ICD. Although this could be a possible explanation behind selective degradation of N1‐ICD by these FBXW7 mutants, the complete rationale underlying D510E and D527G oncogenic activity in ATL still remains elusive. Interestingly, this study strongly advocates the existence of a crosstalk between mutated FBXW7 and several other proteins in ATL cell lines, such as viral oncogene (HTLV‐I Tax), p53, c‐Myc, etc. Given the high rate of mutation in both p53 and c‐Myc in human carcinomas, selective loss of FBXW7 functions may play a more decisive role in the oncogenic process. Furthermore, FBXW7 mutations in ATL tumors may create a window of opportunity for targeted anticancer therapies. For instance, combinatorial therapy using the drug Imatinib along with siRNA‐mediated FBXW7 inactivation in mice has been found to drive the depletion of leukemia‐initiating cells when compared to normal hematopoietic stem cells.^38^

### 3.3 Sézary syndrome

Sezary syndrome (SS) was first identified in 1938 and is also known as “the red man syndrome” or '1' homme rouge'. SS is a cutaneous T‐cell lymphoma in which CD4+ tumor cells also known as Sézary cells are localized in the skin, lymph nodes, and peripheral blood. Clinically, this disease has often been misdiagnosed and managed as chronic dermatitis or psoriasiform lesion. Although SS can be treated with low‐dose prednisone and chlorambucil or by using immunomodulating protocols, the prognosis remains extremely poor with a median survival between 2 and 4 years post‐diagnosis and disease‐specific 5‐year survival of about 15 to 20%. New treatment modalities, which utilize histone‐deacetylase (HDAc) inhibitors along with allogeneic stem cell transplantation, are now being explored to treat SS cases. Over the years, various mechanisms have been proposed with respect to constitutive NOTCH1 signaling activation in SS cells, including activating mutations in the NOTCH1 gene. Sequence analysis of the HD region (exon 26) and PEST region (exon 34) of the NOTCH1 along with the entire coding region of the FBXW7 revealed the absence of any kind of mutations in the HD and PEST domain region of NOTCH1 and/or FBXW7, suggesting that other regulatory mechanisms might be in operation in SS. This is in sharp contrast to what is normally observed in ALL cases where aberrant activation of NOTCH1 is mostly linked to mutations in the HD and PEST domain, or mutations in FBXW7, which impair ubiquitination and proteasomal degradation of N1‐ICD protein. Incidentally, the current study reported no specific mutation within the phosphatase and tensin homolog (PTEN) that is again in contrast with an earlier report that found instances of heterozygous deletion within PTEN, albeit in limited SS cases. Altogether, these observations indicate the presence of a complicated interactome involving NOTCH, FBXW7, and PTEN in SS cells that warrant further investigation.^39^, ^40^

### 3.4 Squamous‐cell cancer of the head and neck

Squamous cell carcinoma of the head and neck (SCCHN) is one of the most common cancer types worldwide and accounts for more than 500,000 new cases and close to 4000,000 deaths per year. SCCHN depicts a heterogeneous disease entity with a range of etiological factors attributed to the pathogenesis of distinct molecular subsets of tumors, which display distinct biological and clinical features. Apart from the well‐known risk factors such as tobacco smoking and alcohol consumption, infection with human papillomavirus (HPV) has now become more critical to understand the epidemiology and prognosis of SCCHN. Most SCCHN cases are diagnosed with locally advanced disease states, which often warrant multimodal treatment strategies. Despite significant improvements in radiation and surgical techniques and the use of chemotherapy and monoclonal antibody protocols in the advanced stage of the disease, more than half of all patients experience a relapse with extremely poor prognosis, not suitable for treatment options with curative intention. Nevertheless, the treatment of SCCHN is expected to change in the future as targeted therapies continue to be explored. Recently, a next‐generation sequencing study conducted on SCCHN specimens revealed a previously unknown insight into the molecular pathogenesis of the disease. The study unraveled novel mutations affecting genes involved in the differentiation program of squamous epithelium, especially in the NOTCH/p63 axis, such as NOTCH1, TP63, and FBXW7. The most remarkable finding emerged from the whole‐exome sequencing studies was the high incidence of loss‐of‐function mutations in the NOTCH1 gene. It was observed that the majority of these NOTCH1 mutations in SCCHN were missense mutations, which were localized at or close to identified important receptor domains such as the ligand‐binding domain (EGF repeats 11, 12, and 13) or the ankyrin region. Moreover, the detected nonsense mutations were found to result in truncated NOTCH1 proteins lacking domains required for transcriptional activity. These available data imply that NOTCH1, owing to certain mutational events, may act as a tumor suppressor in head and neck epithelium. It was further observed that p63 downregulation by NOTCH decreased the ability of NOTCH to limit growth and differentiation along with antagonistic effects on Hes/HERP family members, like Hes‐1, Hey‐1, and Hey‐2. p63 has been reported to modulate the expression of the JAGGED1 and/or 2, thereby assisting NOTCH in expanding its signaling effects to adjacent cells. It appears from the current study that there exists a complex negative feedback loop between NOTCH and p63 that may regulate the balance between self‐renewal and differentiation. Given the negative control of FBXW7 on the NOTCH pathway, it will be interesting to further investigate the FBXW7/NOTCH/p63 axis in SCCHN.^41^, ^42^

### 3.5 Esophageal squamous cell carcinoma

Esophageal squamous cell carcinoma (ESCC) is viewed widely as a serious malignancy with respect to prognosis and mortality rate. Esophageal carcinoma is the eighth most common cancer worldwide in terms of incidence, and sixth most in terms of cancer‐related deaths worldwide with developing nations making up more than 70% of total cases and deaths. Despite numerous advances in diagnosis and treatment, the 5‐year survival rate for all patients diagnosed with esophageal cancer is abysmal. A recent study performed exome sequencing on tumor‐normal pairs that revealed non‐silent mutations both in tumor tissues and cell lines. Genes involved in cell cycle and apoptosis administration were found to be mutated by somatic alterations of tumor protein 53 (TP53), cyclin D1 (CCND1), cyclin‐dependent kinase inhibitor 2A (CDKN2A), nuclear factor erythroid 2‐related factor 2 (NFE2L2), and retinoblastoma transcriptional corepressor 1 (RB1) in almost 99% of studied cases. Moreover, histone modifier genes, including KMT2D (also called MLL2), histone methyltransferase 2C (KMT2C), lysine demethylase 6A (KDM6A), EP300, and CREBBP were found to be mutated. Even further, the Hippo and NOTCH pathways were found to be dysregulated and could be attributed to mutations in AJUBA, FAT1, FAT2, FAT3, or FAT4, and NOTCH1, NOTCH2 or NOTCH3 or FBXW7. Altogether, these findings present the mutational landscape of ESCC and highlight mutations as key epigenetic modulators with prognostic and potentially therapeutic implications.^43^, ^44^

### 3.6 Adenoid cystic carcinoma

Adenoid cystic carcinoma (ACC) is a malignant neoplasm arising from salivary glands of head and neck region. This type of tumor was first described as a benign neoplasm and was named cylindroma owing to the cribriform appearance formed by tumor cells with cylindrical pseudo spaces. Approximately 12 to 15% of all salivary gland tumors are ACCs, which are more common in minor salivary glands and the submandibular gland and less common in the sublingual and parotid glands. ACC is a very rare tumor of the head and neck that accounts for less than 1% of all head and neck cancers. Currently, ACC, especially of the trachea lacks well‐characterized diagnostic markers and there is no specific therapy available against metastatic ACC of the trachea. Genomic mutations have been reported in the NOTCH pathway that has been investigated in order to determine the efficacy of NOTCH inhibitor in ACC of the trachea. For instance, gain‐of‐function mutations of the NOTCH1 gene were found to be quite frequent that leads to stabilization of the N1‐ICD. Furthermore, NOTCH2, NOTCH4, JAGGED1, and FBXW7 mutations have also been identified in some ACC cases. Incidentally, the observed NOTCH mutation can be associated with solid subtype and shorter overall survival but cannot be considered as an independent prognostic factor in the presence of several histologic subtypes. Nonetheless, NOTCH and FBXW7 mutations can be studied further to examine the pathogenesis and prognosis of ACCs.^45^, ^46^

### 3.7 Melanoma

Melanoma is a malignant neoplasm of the melanocytes. Melanocytes are cells embedded in the basal layer of the epidermis that produces the pigment melanin, responsible for skin color. As one among the three major forms of skin cancer, with basal cell carcinoma and squamous cell carcinoma being the other two, melanoma has the worst prognosis, resulting in the vast majority of skin cancer‐related mortalities. Given the high mortality rates, a greater understanding of the molecular pathogenesis and disease‐critical interactions of malignant melanoma is urgently required. FBXW7 has been found to be mostly inactivated in melanoma that results in constitutive NOTCH1 activation. And therefore, FBXW7 mutations may be considered as a “driver” genetic event in malignant melanoma. A recent study utilized an exome sequencing screen to characterize the functional impact of FBXW7 mutations in melanoma, and highlight its substrate NOTCH1 as a suitable therapeutic marker in the clinical setting. shRNA (shRNA1 and shRNA2)‐mediated silencing of FBXW7 using lentiviral transduction of melan‐a melanocytes expressing mutant NRAS (NRASG12D) was found to result in statistically significant upregulation of the NOTCH1 downstream target protein Hey1, with increasing levels observed post‐MG‐132 treatment. Furthermore, the decrease in FBXW7 transcriptional activity was found to promote tumor angiogenesis, observed through enhanced expression of angiogenic driver proteins, including NOTCH1. Based on these findings, it can be said that FBXW7 mutational inactivation represents a potent mechanism for constitutive NOTCH1 activation in melanoma. Additionally, the present study found NOTCH1, c‐Myc, and Cyclin E to be consistently regulated by FBXW7 in melanoma as opposed to Aurora A (inconsistent) and myeloid cell leukemia sequence‐1 (MCL1) (unregulated). Taken together, the current study offers an interesting avenue for anti‐NOTCH treatment strategies in melanoma as well as an understanding of the spectrum of genomic alterations represented within the melanoma tumors, which can be used for stratifying patients for treatment based on these aberrations.^17^, ^47^

### 3.8 Osteochondromas

Osteochondromas are normally bone protuberances surrounded by a cartilage layer that tends to affect the extremities of the long bones in an immature skeleton and deform them. They normally occur singly but multiple forms of presentation may also be seen in certain cases. Osteochondromas have a very typical appearance and are easily diagnosed but an atypical site (in the axial skeleton) and/or malignant transformation of the lesion may sometimes make it challenging to identify osteochondromas immediately using radiographic techniques. There exists a continuing debate on whether osteochondroma is actually a developmental disorder (pseudotumoral lesion) or neoplasm. Nevertheless, irrespective of whether it is a pseudotumoral lesion or a more common benign bone tumor, it is definitely an exostosis phenomenon (external bone proliferation that deforms the bone). Exostosin glycosyltransferase 1 (EXT1), an endoplasmic reticulum transmembrane protein that frequently mutated in multiple osteochondromas, acts as a connector between NOTCH1 and FBXW7. Interaction between NOTCH1 and FBXW7 has been found to be significantly enhanced in the presence of EXT1. Moreover, N1‐ICD level has been found to be significantly reduced in the presence of EXT1. Interestingly, the reduced N1‐ICD levels in the presence of EXT1 were found to be dependent on FBXW7. As a matter of fact, there are about 479 identified mRNAs that are co‐regulated by both EXT1 and FBXW7, which incidentally represent more than 30% of all FBXW7 substrates. It is possible that EXT1 is functionally associated with FBXW7, presumably through priming kinases and substrates like N1‐ICD and therefore, mutational events affecting the EXT1 or FBXW7 gene can significantly affect the pathogenesis in osteochondroma patients through the NOTCH pathway.^48^, ^49^

## 4 INTERACTION BETWEEN ENZYMES MODULATING PHOSPHORYLATION RELATED EVENTS, FBXW7 AND NOTCH

### 4.1 Kinase

Targeted inhibition of the NICD, which is an essential component of the NOTCH pathway, is now widely being explored as a novel strategy for treating a wide range of cancer. The activity or stability of NICD is driven by numerous protein‐protein interactions and post‐translational modifications (PTMs), such as ubiquitination, phosphorylation, etc. Among all these interactions, NOTCH ubiquitination and degradation by FBXW7 is considered to be one of the most relevant. As mentioned earlier, FBXW7 directly binds NICD to facilitate its ubiquitination and subsequent proteasomal degradation. These FBXW7‐mediated processes essentially require phosphorylation at Thr‐2512 residue of the NICD. Until now, only two kinases were known to phosphorylate NOTCH1 at Thr‐2512, namely the homeodomain‐interacting protein kinase 2 (HIPK2) and mitogen‐activated protein/ERK kinase kinases1 (MEKK1). Recently, dual‐specificity tyrosine‐phosphorylation‐regulated kinase 2 (DYRK2), a Ser/Thr kinase was reported to play a major role in the regulation of cellular proliferation, differentiation, and survival. DYRK2 was found to interact with a panel of signaling pathways through the phosphorylation of specific proteins, such as eIF2Bε, CRMP4, 4E‐BP1, NFAT, p53 tau, hPXR, c‐Jun, c‐Myc, GSK, Snail, katanin, and Siah2. DYRK2 aberrations have been reported in the development and progression of several human carcinomas, such as esophageal adenocarcinoma, breast cancer, non‐small cell lung cancer, ovarian serous adenocarcinoma, etc. DYRK2 has been shown to limit epithelial‐mesenchymal transition (EMT) by degrading Snail in ovarian cancer and its knockdown promotes tumor growth and invasion, thereby attesting its role as a tumor suppressor. Furthermore, in response to genotoxic stress, the protein ataxia‐telangiectasia mutated (ATM) has been shown to phosphorylate and stabilize DYRK2 that in turn phosphorylates p53 at Ser46, thereby driving cellular apoptosis. DYRK2 has also been reported to act as an upstream negative regulator of the NOTCH pathway that is highly active in several cancers. DYRK2 can directly associate and phosphorylates the NICD at Thr‐2512, thereby facilitating its proteasomal degradation, driven by the E3 ligase FBXW7. RAM domain together with the protein MAML1 helps co‐localize NICD and CBF1, Suppressor of Hairless, Lag‐1 (CSL) in order to activate the transcription of NOTCH downstream target genes. These reports suggest that the RAM domain plays a key role in promoting the interaction between N1‐ICD and DYRK2 and therefore, DYRK2 modulation by chemotherapeutic agents may have a strong effect on the viability, motility, migration, and invasion capacity of carcinomas expressing NOTCH1.^50^

Another key positive regulator serum/glucocorticoid‐inducible kinase 1 (SGK1) has been identified to interact with NOTCH through FBXW7. SGK1 is a vital regulator of an array of cellular processes, including metabolism, channel conductance, cell volume, differentiation, proliferation, and survival. SGK has a very close sequence identity with the catalytic domain of Akt and with the phosphorylated serine and threonine residues that rest in the RxRxx[S/T] motif. SGK1 is regulated in a phosphoinositide 3‐kinase (PI3K)‐dependent fashion in response to insulin or growth factor signaling. Once activated, SGK1 phosphorylates an array of substrates, such as GSK‐3β, b‐Raf, IKKβ, FKHRL1, Nedd4‐2, p27, Fe65, and SEK1. SGK1 also acts as an inhibitor of γ‐secretase enzyme that is necessary for NOTCH1 pathway activation. A recent study by Mo et al. found that N1‐ICD is able to associate in a trimeric complex along with FBXW7 and SGK, suggesting that SGK1 may facilitate the degradation of N1‐ICD through an FBXW7‐dependent mechanism. In fact, activated SGK1 assists in decreasing N1‐ICD stability by activating FBXW7 through phosphorylation at serine 227 position. Accumulated dexamethasone in cells can activate SGK1 that in turn activates FBXW7 through serine phosphorylation as mentioned. In line with this observation, both the mRNA and protein level expression of N1‐ICD has been found to be enhanced in SGK1‐deficient cells when compared to SGK1 wild‐type cells. Collectively these results show that the kinase SGK1 can inhibit the NOTCH1 signaling by hastening the degradation of N1‐ICD by FBXW7. However, till date, the mechanism by which SGK1 controls the anti‐leukemic effect of glucocorticoids has remained unknown and for that reason the SGK1/FBXW7/N1‐ICD trimeric complex may represent a potential therapeutic target for improving the efficiency of anti‐leukemic therapies in T‐ALL tumors expressing NOTCH1.^51^

It must be noted that FBXW7 explicitly recognizes phosphorylated substrates. GSK‐3β is a key kinase involved in the FBXW7 pathway. There is enough data to suggest that the sequences identified by FBXW7 in c‐Myc, c‐Jun, and SREBP1a or ‐1c are actually phosphorylated by GSK‐3β, which drives the ubiquitylation and degradation of these substrates through the FBXW7 pathway. Although N1‐ICD is known to be phosphorylated by GSK‐3β, the repression of endogenous GSK‐3β activity in mammalian cells surprisingly does not increase but rather decreases the stability of N1‐ICD. Moreover, genetic investigations in Drosophila have found GSK‐3β homolog Shaggy to be involved in the positive regulation of the NOTCH signaling cascade. These observations indicate that GSK‐3β‐mediated phosphorylation of N1‐ICD may not necessarily trigger its recognition and degradation by FBXW7. A very recent study identified a NOTCH phosphodegron that was somewhat different from the “standard” phosphodegrons of c‐Myc and Cyclin E. Interestingly, the serine or threonine residue at position +4 corresponding to the central threonine was replaced with glutamic acid in the NOTCH phosphodegron motif. This subtle difference in the N1‐ICD phosphodegron motif may be responsible for driving the GSK3‐mediated kinase activities, thereby regulating diverse cellular processes in a cell type‐ or developmental stage‐specific manner.^18^, ^52^

### 4.2 Phosphatase

Serine/threonine/tyrosine interacting protein (STYX), a protein belonging to the tyrosine phosphatases (PTPs) family, is by basic functionality a pseudophosphatase. Pseudophosphatases carry mutations within their active site signature motif (HCX5R) that makes them inactive. They resemble substrate trapping mutants, essentially PTPs mutated to bind their substrates, as “wild type” substrate binding mutants. Although pseudophosphatases preserve the three‐dimensional fold and their ability to bind phosphorylated proteins, however, in comparison to PTPs, pseudophosphatases can't dephosphorylate the phosphorylated protein during cellular processes. STYX has a diffused expression in various tissues but not much is known about its biological role in cancers. STYX has been associated with cell proliferation, migration, invasion, and apoptosis in colorectal cancer cells and a range of studies have indicated that STYX acts as a latent oncogene that inhibits apoptosis in colorectal and breast cancer, mainly by binding to FBXW7. In breast cancer cells, STYX has been found to suppress FBXW7 expression through direct protein‐protein binding. Furthermore, in endometrial cancer (EC) cells, STYX has been found to interact with FBXW7 in regulating the NOTCH and mTOR signaling pathway. In endometrial tumors, aberrant activation of the NOTCH cascade has been associated with increased cell proliferation, and suppression of the mTOR pathway has been found to repress tumor initiation and progression. A recent study found that FBXW7 regulates the proliferation and apoptosis of EC cells by initially suppressing the NOTCH signaling linked protein and p‐mTOR/mTOR, and subsequently the NOTCH/mTOR signaling pathway. Interestingly, in EC cells overexpressing FBXW7, STYX was found to directly interact with FBXW7 and also assisted in balancing the FBXW7 expression levels. Consistent with this observation, FBXW7 overexpression was found to promote proliferation and limit apoptosis of EC cells in a STYX‐dependant fashion, and the deployment of NOTCH activator JAGGED1 together with mTOR activator MHY1485 reversed the function of overexpression of FBXW7 in the context of cell proliferation and apoptosis. Furthermore, the NOTCH inhibitor DAPT was found to reverse the function of the overexpression of STYX. Collectively, these observations indicate that the STYX/FBXW7 axis is involved in the development of EC cells and may be involved in the development of other tumors as well through the regulation of NOTCH‐mTOR signaling pathway. And therefore, STYX being active in several cancers, its role in modulating the NOTCH‐mTOR interaction through FBXW7 warrants further attention.^53^, ^54^

### 4.3 Peptidyl‐prolyl cis‐trans isomerase

Prolyl‐isomerase (Pin1) is the only recognized peptidyl‐prolyl cis‐trans isomerase (PPIase) that explicitly recognizes and isomerizes the phosphorylated Serine/Threonine‐Proline (pSer/Thr‐Pro) motif. In other words, Pin1 catalyzes the cis/trans conversion of specific motifs found in certain proteins, thereby triggering a conformational change necessary for the complete activity and crosstalk of a range of signaling pathways. Pin1 has been attributed to multiple cellular processes, the aberration of which can result in both degenerative and neoplastic outcomes. Pin1 is highly expressed in the bulk of cancers and its inhibition has been found to significantly repress cancer progression. Recently, Pin1 was found to be essential for sustained NOTCH activity in breast cancer cells irrespective of FBXW7α status. In fact, isomerization triggered by interaction with Pin1 was held accountable for the recognition and degradation of N1‐ and N4‐ICD by FBXW7α in a PP2A‐dependent fashion. These observed interactions between Pin1 and NOTCH1/4 is extremely relevant in modulating the NOTCH paralog‐driven functions in breast tumors. NOTCH4 is an essential modulator of basal breast cancer stem cell (BCSCs) population, while NOTCH1 activity is more limited to luminal precursors both in the normal mammary gland as well as in breast tumors. The recent findings now place Pin1 as a key facilitator of stage‐specific physiological and pathological NOTCH functions in the breast epithelial compartment. FBXW7α has been implicated in the maintenance of normal neural, colon, and hematopoietic stem cells primarily through the degradation of NICD. This study shows that FBXW7 acts as a key negative driver acting against the proliferation of the BCSCs compartment, through the degradation of N1‐ and N4‐ICD, thereby preventing BCSC self‐renewal and metastasis *in vivo*. Nevertheless, the role of Pin1 is extremely significant in maintaining high levels of N1‐ICD and N4‐ICD, irrespective of FBXW7α expression. Based on these observations, it is therefore likely that aberrant expression or activity of Pin1 may strongly diminish the selective pressure for activating NOTCH or FBXW7α mutations.^55^, ^56^

## 5 NOTCH AND FBXW7‐BASED DEREGULATIONS IN MODULATING KEY ONCOGENIC PROCESSES

### 5.1 Cell cycle, differentiation, and proliferation

Deregulation of the cell cycle, which is a characteristic feature of oncogenic transformation, promotes genetic instability and drives abnormal cell proliferation. In the past decade, the domain of cancer genetics has shown that hyperactivating mutations in embryonic signaling networks, such as NOTCH, Wnt, and Shh, coupled with loss of function of tumor suppressor proteins drive malignant transformation of a range of tumors. Gene expression profiling of these complicated and redundant mitogenic processes to recognize prognostic signatures and their therapeutic targeting have proved challenging to the core. The cell cycle that acts as an integration point for an array of information cascaded through the upstream signaling pathways depicts a viable target for diagnostic and therapeutic modalities. As a result, analysis of the aberrant mitotic engine proteins and associated deregulations in human carcinomas are now paving the way for the identification of novel cell cycle‐directed therapies. The mammalian cell cycle is a highly organized and regulated mechanism that guarantees perfect duplication of genetic material through cell division. This subtly controlled regulation is contingent on growth‐regulatory stimulus as well as signals by proteins monitoring the genetic integrity to ensure that there is not any genetic damage whatsoever. Carcinogenesis is characterized by aberrant cell cycle activity that leads to uncontrolled cell proliferation and subsequent deregulation of the apoptotic machinery, thereby preventing normal cell death.^57^, ^58^, ^59^ In this section, we present the recent advances made with respect to the role of FBXW7 and NOTCH in driving aberrant cell cycle activity in tumors that might be of therapeutic significance in identifying targeted therapies.

FBXW7 mediates the ubiquitylation and sequential degradation of proteins, such as Cyclin E, c‐Myc, c‐Jun, and NOTCH, which regulate cell cycle activity. As discussed earlier, a wide range of tumors harbor loss‐of‐function mutations in the FBXW7 gene, which ultimately result in the disproportionate accumulation of FBXW7 substrates with carcinogenic outcomes. For instance, unexpected loss of FBXW7 activity in mouse embryonic fibroblasts (MEFs) has been reported to induce cell cycle arrest and prevent apoptosis, primarily driven by abnormal aggregation of N1‐ICD. Forced expression of NICD1 reverses the phenotypes of FBXW7‐deficient (FBXW7Δ/Δ) MEFs with respect to cell cycle and apoptosis. A recent study found that Rbpj deletion normalizes the aberrant cell cycle activity as seen in FBXW7Δ/Δ MEFs, thereby attributing NOTCH1 signaling cascade in the process. In contrast, the conditional inactivation of the p53 gene (that operates largely as a transcription factor, and can trigger a variety of antiproliferative programs) was found to prevent cell cycle arrest but did not trigger apoptosis in FBXW7Δ/Δ cells. Altogether, these findings imply that FBXW7 may not directly function as an onco‐suppressor in MEFs, rather it plays a more supportive role in cell cycle progression and cell survival, primarily through regulation of N1‐ICD. Furthermore, increased cell cycle activity and decreased apoptosis as observed in FBXW7Δ/Δ MEFs can be attributed to NOTCH hyperactivation, and p53 may be involved in the process, thereby signifying the pathological importance of FBXW7/NOTCH/p53 axis during carcinogenesis.^18^, ^60^ There are similar studies that enforce the hypothesis that FBXW7/NOTCH/p53 axis may be crucial in driving cell cycle‐associated activities in tumors. In human hepatocellular carcinoma (HCC), RBP‐J‐interacting and tubulin‐associated (RITA) has been found to facilitate the nuclear export of RBP‐J to tubulin fibers that in turn downregulates NOTCH‐mediated transcription. Moreover, RITA overexpression in HCC cell lines increases the expression levels of p53 and FBXW7 while limiting the expression of key cell cycle mediators, such as Cyclin D1, Cyclin E, Cdk2, Hes‐1, and NF‐κB p65. These changes ultimately trigger G0/G1 cell cycle arrest, inhibit growth, and promote apoptosis in SMMC7721 and HepG2 cell lines. It can be understood that RITA exercises its tumor‐suppressive effects primarily through induction of G0/G1 cell cycle arrest and apoptosis, thereby indicating the functional significance of the FBXW7/NOTCH/p53 axis not only in HCC but possibly in other tumors as well.^61^, ^62^

Family with sequence similarity 83, member D (FAM83D) is a microtubule‐associated protein (MAP) with dynamic mitotic function that has been found to be deregulated in several tumors. Predominantly, FAM83D localizes within the cytoplasm during interphase and at spindle microtubules and poles during mitosis. FAM83D transcription is upregulated and the protein phosphorylated upon the cell entering the G2/M phase. FAM83D depletion in cells helps break the chromosomes aggregation on the metaphase plate and on the nuclear envelope breakdown (NEBD), thereby delaying the start of anaphase during mitosis. FAM83D overexpression has been extensively reported in primary tumors carrying TP53 mutations when compared to those with wild‐type TP53 gene. Together, these observations indicate that FAM83D may have a key role to play during tumor cell growth and proliferation, primarily by exerting its control on cell cycle activity. A recent study investigated the role of FAM83D in human colorectal cancer and found that FAM83D mRNA expression level was significantly upregulated in tumor cells when compared to adjacent normal colon cells. Similar observations were reported in human colorectal cancer cell lines, such as DLD‐1, Caco‐2, RKO, HT‐29, LoVo, SW480, and HCT116, where both FAM83D mRNA and protein level expression were found to be upregulated, especially in SW480 and HCT116 lines. FAM83D knockdown in HCT116 and SW480 was found to be closely associated with decreased cell proliferation, colony formation, migration and invasion, and increased apoptosis of these cell lines. Interestingly, FAM83D knock‐down also resulted in the upregulation of FBXW7 and an expected downregulation of N1‐ICD. Introduction of FBXW7 siRNA completely reversed the suppressive effect of FAM83D knockdown on NOTCH1, thereby suggesting an important role of FAM83D in modulating NOTCH activity through the E3 ligase, FBXW7. Moreover, forced NOTCH1 overexpression in FAM83D knockdown cells was found to have a profound impact on the cell proliferation, migration, and invasion of HCT116 and SW480 cell lines. Collectively, these pieces of evidence suggest that FAM83D may be involved in the FBXW7/NOTCH1 degradation pathway and therefore, may be explored as a molecular target for colorectal cancer diagnosis and treatment.^63^, ^64^

FBXW7 has been found to modulate the differentiation and proliferation of keratinocytes by facilitating the degradation of key proteins like c‐Myc and NOTCH. A recent study reported that FBXW7‐deficiency in keratinocytes increases the proliferative capacity, mainly through the accumulation of c‐Myc but not NOTCH. However, FBXW7 deficiency drives early differentiation of keratinocytes in a manner that is dependent on both NOTCH and c‐Myc. Although FBXW7‐deficient keratinocytes proliferate excessively *in vitro*, loss of FBXW7 does not activate keratinocytes to differentiate into squamous cell carcinoma (SCC) *in vivo*, triggered by the expression of oncogenic Ras. A plausible explanation behind this could be that the keratinocytes stem cell population becomes exhausted as a result of heightened NOTCH activity. These interesting findings show that FBXW7 may be involved in regulating proliferation and differentiation of keratinocytes through inhibitory and stimulatory actions during skin carcinogenesis. In fact, FBXW7 could be discharging two conflicting duties during skin carcinogenesis by promoting or inhibiting tumor formation through selective degradation of NOTCH and c‐Myc, respectively. As mentioned earlier, NOTCH accumulation in FBXW7‐deficient keratinocytes suppresses the tumor formation driven by oncogenic H‐Ras. In line with this, genetic suppression of NOTCH activity coupled with activation of Ras in keratinocytes has previously been shown to foster the development of aggressive SCC that can be partially attributed to the rise in the expression levels of proteins, such as MRCKα, ROCK1, and ROCK2, all of which operate downstream of Rho‐family GTPases. NOTCH1 downregulates the expression of these kinases, and tumorigenesis is completely abolished through their inhibition. In FBXW7‐deficient keratinocytes, the abundance of kinases, like MRCKα, ROCK1, and ROCK2, has been found to be significantly downregulated. Moreover, forced deletion of Rbpj reverses these effects and enhances tumor formation. Therefore, it can be said that FBXW7 likely controls keratinocyte behavior by modulating the NOTCH‐dependent regulation of small‐GTPase signaling pathways. Given that the complex crosstalk between NOTCH and multiple oncoproteins including c‐Myc is a key determinant of homeostasis in epithelial tissues, FBXW7 appears to be pivotal molecule involved in the regulation of differentiation and proliferation of epidermal stem cells and tumorigenesis in epithelial tissues. This hypothesis may hold true for other types of tumor cells as well, excluding the epithelial cohort. It can be conferred that reduced cell proliferation due to overaccumulation of NOTCH and/or c‐Myc in many tumor types may involve selective degradation by FBXW7 in a tissue‐specific manner.^14^, ^27^ Intestinal FBXW7 inactivation can induce adenomas and its inactivation in intestinal cells has been found to deregulate the NOTCH and Jun pathways, which affects the fate of progenitor cells and proliferating crypt cells. DEK, a highly conserved nuclear factor that is preferentially expressed in actively proliferating and malignant cells, is a prime target of GSK‐3β‐mediated phosphorylation, and is responsible for switching the alternative RNA splicing of tropomyosin (TPM) isoforms. FBXW7 inactivation prevents the degradation of the DEK, which leads to selective loss of the epithelial isoform of TPM that along with NOTCH and c‐Jun, drives carcinogenesis in mice models. As would be expected, FBXW7 inactivation leads to the upregulation of both NOTCH and c‐Jun, which are FBXW7 substrates. NOTCH signaling has been implicated in the regulation of cell fate decisions, mainly through its downstream effector Hes‐1. Although some reports suggest that Hes‐1 and Hes‐5 both participate in the NOTCH‐mediated cell fate determination, evidence supporting the role of Hes‐5 is not conclusive enough. Nevertheless, overexpression of Hes‐1, and not Hes‐5 in intestinal tumors, indicates that these may be regulated by additional transcription factors and therefore, have overlapping yet discrete patterns of expression in the gut. Hes‐1, −6, and − 7 transcripts are mostly restricted to the crypt region, while Hes‐5‐expressing cells have been reported in the villus epithelium and the mesenchyme. Collectively, these observations suggest that FBXW7 mutation can drive failed intestinal differentiation and promote proliferation through NOTCH downstream effectors and possibly DEK. Consistent with this paradigm, an intact and functional FBXW7 may therefore restore homeostatic control mechanisms and impair oncogenic activators by facilitating their steady degradation. Therefore, therapeutic modalities based on the partial delivery of FBXW7‐α to intestinal tumor cells may limit nuclear DEK and β‐catenin accumulation and drive better prognosis in patients.^15^, ^65^

### 5.2 Apoptosis

Apoptosis is the mechanism of programmed cell death that is driven by energy‐dependent biochemical mechanisms and characterized by distinct morphological features. It is an essential component of several biological processes including normal cell turnover, hormone‐dependent atrophy, proper development, functioning of the immune system, etc. Aberrant apoptotic mechanism (either too little or too much) has been attributed in a range of human conditions, such as neurodegeneration, ischemic injury, autoimmune disorders, and different types of cancer. The distinct ability to regulate the life or death of a cell is considered a modality with immense therapeutic potential. Hence, research continues to focus on the identification and interpretation of the cell cycle machinery and associated signaling cascades that modulate cell cycle arrest and apoptosis. Although a panel of key apoptotic proteins has been identified, the precise molecular mechanisms of operation of these proteins remain a challenge. A wide range of anticancer therapies currently deployed in clinical oncology exploit the unimpaired apoptotic signaling cascades to initiate cancer cell death. Therefore, an aberration in the apoptotic pathways may drive drug resistance in tumors, so limiting the effectiveness of therapies. Accordingly, a greater understanding of the apoptotic cell death signaling pathways and associated protein‐protein interactions may augment the efficacy of cancer therapy and help in bypassing drug resistance. Several proteins both pro‐apoptotic and anti‐apoptotic participate in the cell death process, such as Bcl‐2, Bcl‐xL, p53, Mcl‐1, etc.^66^, ^67^In addition to driving cell growth, proliferation, and differentiation, overexpression of c‐Jun, c‐Myc, or NOTCH can also trigger programmed cell death or apoptosis. However, uncertainty surrounds around how FBXW7‐deficient cells elude apoptosis in a setting with upregulated c‐Jun, c‐Myc, and/or NOTCH levels. One study suggests that FBXW7 regulates cellular apoptosis by targeting MCL1, a pro‐survival Bcl‐2 family member for ubiquitylation and degradation in a GSK3‐dependent manner. Human T‐ALL cell lines display a close association between FBXW7 deficiency and MCL1 overexpression and therefore, T‐ALL cell lines with defective or inactive FBXW7 are resistant to Bcl‐2 antagonist ABT‐737 but are sensitive to the multi‐kinase inhibitor, Sorafenib. FBXW7 activation or MCL1 deregulation has been found to revive the sensitivity of T‐ALL cell to ABT‐737, thereby positioning MCL1 as a therapeutically important bypass survival protein that empowers FBXW7‐deficient cells to evade apoptosis. Furthermore, there are studies that have found a direct interaction between NOTCH and MCL1 in the regulation of programmed cell death. For instance, NOTCH1 and NOTCH2 signaling are constitutively activated in chronic lymphocytic leukemia (CLL) tumors, and inhibition of NOTCH1 and NOTCH2 has been found to enhance apoptosis in CLL cells, which is accompanied by downregulation of MCL1 and upregulation of IL4. A limited set of studies argue that MCL1 downregulation by NOTCH may not be due to decreased transcription or degeneration by caspases but due to enhanced degradation through a FBXW7‐dependent process. Collectively, these findings position FBXW7, NOTCH, and MCL1 as key drivers of apoptosis.^68^, ^69^

### 5.3 Metastasis

Metastasis remains a major concern and a principal cause of death in cancer patients. Therefore, elucidation of the genes and mechanisms that control this process has remained an area of active research. Metastasis essentially involves a complex array of a process that begins with the detachment of cancer cells from a primary tumor followed by their migration to neighboring tissue, entry into the circulatory system, and finally, invasion into distant organs to form secondary tumors. As discussed extensively, FBXW7 mutations are frequent in T‐ALL (31%) and cholangiocarcinoma (35%). A majority of these studied mutations are missense in nature and a result of amino acid substitutions at key arginine residues such as Arg465 and Arg479 in the WD40 region of FBXW7. Several studies have found FBXW7 deregulation in the host environment as one of the key determinants of cancer metastasis. For instance, Yumimoto et al. characterized the mechanism underlying metastasis of bone marrow stromal cells (BMSCs) and found that FBXW7 deletion results in NOTCH accumulation and subsequent activation of the protein CCL2. In fact, CCL2 expression in FBXW7‐deficient cells overexpressing NOTCH drives the formation of metastatic niches through the localization of Monocytic Myeloid‐Derived Suppressor Cells (Mo‐MDSC). Therefore, inhibition of the CCL2/CCR2 pathway limits the metastatic incidence observed in FBXW7‐deficient mice. CCL2 has also been attributed in the recruitment of monocytes/macrophages to sites of pulmonary metastasis in mice with breast tumors. CCL2 is mainly expressed by BMSCs, along with other cells facilitating tumor metastasis such as bone marrow‐derived dendritic cells (BMDCs), fibroblasts, endothelial cells, smooth muscle cells, etc. These observations suggest that the interaction between FBXW7, NOTCH, and CCL2 can be a crucial driver event of cancer metastasis. Moreover, identification of FBXW7 and NOTCH as upstream regulators of CCL2 expression provides an insight into the mechanism by which the production of this chemokine is regulated. This potentially could be a basis for the development of new treatment strategies aiming to target the metastatic phenomenon in tumors.^70^, ^71^

Alpha‐synuclein (α‐synuclein or SNCA), a soluble presynaptic protein belonging to the synuclein protein family, is known to form insoluble fibril aggregates, which play a very important role in the pathogenesis of Parkinson's disease (PD), however, its precise role in carcinogenesis is not clearly understood. Studies have found that α‐synuclein is abundantly expressed in primary and metastatic melanomas but remain largely untraceable in normal skin and non‐melanocytic cutaneous carcinoma. This can hold true for other metastatic carcinomas as well, such as brain tumors where α‐synuclein is expressed handsomely when compared to benign meningiomas. α‐synuclein can differentially modulate the production of melanin in melanoma and dopaminergic neuronal cells, thereby affecting cell susceptibility to ultraviolet radiation‐induced injury. Nevertheless, the pathological relevance of α‐synuclein during the malignant progression of carcinomas is still being investigated. A recent study reported a previously unknown function of SNCA in facilitating the degradation of N1‐ICD through the FBXW7 pathway. Principally, SNCA represses NOTCH1 transcription at the genetic level and also limits the interaction between N1‐ICD and RBP‐Jk. Moreover, both exogenous and endogenous SNCA downregulates N1‐ICD protein levels in tumors mainly through the proteasomal pathway rather than the lysosomal pathway, thereby suggesting that SNCA might be serving as a connecting link between FBXW7 and N1‐ICD. There are studies that support this hypothesis and have found SNCA to directly interact with FBXW7. Altogether, given the abundance of SNCA in metastatic melanomas and possibly in other tumors, these findings indicate that SNCA may be a key regulator of metastasis through the FBXW7/NOTCH axis.^25^, ^72^

### 5.4 Other miscellaneous key oncogenic events

Notch hyperactivation may be caused by mutations of NOTCH‐associated genes, altered upstream key oncogenic events, or microenvironment signals. Cancer cells may exploit this aberrant signaling to “educate” the surrounding microenvironment cells toward a pro‐tumoral behavior. For instance, in context to tumor microenvironment (TME), it has been observed that epithelial NOTCH1 signaling drives metastasis in serrated colorectal cancer (CRC), where poor‐prognosis of CRC subtypes CMS4/CRIS‐B are controlled by NOTCH1. Furthermore, TGF‐β‐mediated neutrophil infiltration has been found critical for NOTCH1‐driven metastasis, although neutrophil targeting provides therapeutic opportunity in metastatic CRC. In addition, NOTCH1 signaling can be activated via mutation of *FBXW7*, found in 11% of human CRCs. Thus, epithelial NOTCH signaling rewires the TME of CRC to drive poor‐prognosis subtypes and metastasis.^73^ Recently, the role(s) of NOTCH signaling in T‐cell antitumor immunity and TCR‐ and chimeric antigen receptor‐based immunotherapies has been reported. NOTCH signaling is required for optimal T‐cell‐mediated antitumor immunity. Consequently, tumor cells and the TME have acquired mechanisms to suppress NOTCH signaling to evade T‐cell‐mediated killing. Tumor‐mediated suppression of NOTCH signaling in T‐cells can be overcome by systemic administration of NOTCH agonistic antibodies and ligands or proteasome inhibitors, resulting in sustained NOTCH signaling and T‐cell activation. In addition, NOTCH receptors and ligands are being used to ameliorate the generation and specificity of T‐cells for adoptive transplant immunotherapies.^74^ Based on these few evidences, it is concluded that NOTCH and FBXW7 directly or indirectly involve in cancer metabolism, where TME and immune cells play a significant role.

## 6 MULTIPLE DRUG RESISTANCE (MDR), FBXW7, AND NOTCH IN TUMORS

Multidrug resistance (MDR) phenomenon is observed against many effective anticancer drugs and remains a challenge in therapeutic oncology. MDR can develop due to various biological mechanisms, such as activation of detoxifying systems, increased drug efflux and decreased drug uptake, activation of DNA repair mechanisms, evasion of drug‐induced cell death, etc. Although tremendous progress has been made to understand the precise MDR mechanisms *in vitro*, the transfer of this knowledge to the clinical setting has not been met with much success. Hence, identifying genes and pathways crucial to the development of MDR *in vivo* and subsequently establishing reliable protocols for analyzing clinical samples can help design targeted therapies to bypass it. Drug activation *in vivo* involves intricate mechanisms during which drug molecules interact with a host of proteins. These interactions can degrade, modify, or orient the drugs with other proteins and pathways, eventually leading to their activation *in vivo*. Many anticancer drug molecules inevitably must undergo this type of metabolic activation to achieve pure clinical efficacy. Nevertheless, cancer cells over a period of time can develop resistance to such drug molecules and limit their activation. For instance, cytarabine (AraC), a nucleoside drug that is used in the treatment of acute myelogenous leukemia (AML), gets activated after multiple phosphorylation events to transform into AraC‐triphosphate. However, genetic mutations or deregulations of proteins in the activation pathway can limit the activation of AraC, thereby leading to AraC resistance. Several proteins and a panel of associated pathways have been implicated in MDR, such as cytochrome P450 (CYP) system, glutathione‐S‐transferase (GST) superfamily, uridine diphospho‐glucuronosyltransferase (UGT), etc.^75^, ^76^ Several studies have implicated both NOTCH and FBXW7 in the MDR process. The expulsion of CSCs or EMT type cells, which are typically drug‐resistant, is extremely crucial as these are deemed to be the “root cause” of tumor relapse. NOTCH regulates the fate of CSCs and contributes to the acquisition of the EMT phenotype that is involved in driving the MDR phenomenon in a subset of tumors. FBXW7 inactivation has been shown to promote resistance in tumors against certain anti‐tubulin chemotherapeutic agents. Moreover, loss of FBXW7 has been found to be a key prognostic factor promoting resistance to gamma‐secretase inhibitors (GSIs) in certain cancer types. These encouraging reports suggest that targeting NOTCH and/or FBXW7 may be used as a strategy for overcoming MDR. In the subsequent sections, we highlight the biological significance of NOTCH and FBXW7 deregulation in promoting MDR in tumors.^4^, ^77^, ^78^

### 6.1 GSI resistance in tumors

GSIs block NOTCH activation and therefore, present an attractive therapeutic modality for tumors with hyperactive NOTCH signaling. Despite the promise, several tumors become resistant to GSIs over a period of time and continue to grow normally post‐treatment. Some studies have reported FBXW7 mutations in GSI‐resistant tumors, which lead to constitutive activation of the NOTCH pathway. For instance, FBXW7 mutations in T‐ALL tissues may give rise to dominant‐negative FBXW7 alleles, which hamper NICD degradation through the proteasomal pathway. Although FBXW7 mutations may confer resistance to GSIs, NOTCH‐PEST domain truncations in T‐ALL tumors essentially do not have the same effect. One suitable explanation can be that in T‐ALL tumors with PEST mutations, only one allele is affected by the heterozygous mutations while the other allele continues to encode an intact NOTCH. Therefore, increased NOTCH activity arising out of these single‐allele PEST mutations may not be alone sufficient to drive resistance against GSIs. However, FBXW7 double mutations completely block FBXW7 activity, thereby providing a free hand to NOTCH, c‐Myc, and several other substrates in facilitating the T‐ALL resistance against GSIs. c‐Myc being a downstream target of the NOTCH pathway, it may be possible to overcome GSI resistance in T‐ALL tumors by combining GSIs with compounds that block c‐Myc activity. Recently, TMPyP4, a cationic porphyrin that binds to and stabilizes guanine quadruplexes in DNA was found to have beneficial effects when combined with GSIs. Incidentally, c‐Myc harbors a promoter sequence that can form a guanine quadruplex and TMPyP4 is able to bind and restrict c‐Myc transcription and further prevent the growth of tumor cells *in vivo*. Therefore, compounds like TMPyP4 may be beneficial in combination with GSIs to overcome drug resistance in patients expressing FBXW7 double mutations.^79^, ^80^ miRNAs have also been implicated in promoting GSI resistance in T‐ALL tumors through an interaction with both NOTCH and FBXW7. Notably, the NOTCH‐mediated activation of miR‐223 transcription has been found to represses the tumor‐suppressive effects of FBXW7 in T‐ALL cell lines. miR‐223 promoter harbors a conserved RBPjk binding site that is nested within an NF‐kB consensus sequence, thereby suggesting that both NOTCH and NF‐kB act cooperatively to provide regulatory signals to control miR‐223 transcriptional activity. In line with this observation, NOTCH1, NOTCH3, and p65 have all been found to be directly recruited to the miR‐223 promoter site. Therefore, in T‐ALL cells, NF‐kB inhibition has been found to limit the expression of endogenous miR‐223. Consistent with this, the abrogation of the NF‐kB pathway in Jurkat IKKγ−/− cells along with low NOTCH1 and NOTCH3 levels results in the downregulation of miR‐223 mRNA levels. Interestingly, ectopic modulation of miR‐223 expression was found to limit T‐ALL resistance to GSI treatment. Upregulated miR‐223 driven GSI resistance has been attributed in part to FBXW7 and as the data suggest, resistance in Molt3 and Jurkat T‐ALL cells may be due to GSI‐induced increased C/EBPα expression that leads to a completed loss in FBXW7 activity. Altogether, these observations indicate that specific inhibition of miR‐223 may help restore GSI sensitivity in GSI‐resistant T‐ALL cells carrying wild‐type FBXW7 and therefore, can be explored as an attractive targeted therapy for GSI‐resistant T‐ALLs harboring wt FBXW7 and overexpressing miR‐223.^81^

### 6.2 Resistance to chemotherapeutic agents other than GSIs

Prolyl‐isomerase (Pin)‐1 and NOTCH1 have been associated with chemoresistance that is mostly driven by the activity of CSCs. In particular, NOTCH1 plays a key role in this process since it facilitates the transcription of a panel of cell survival and drug efflux genes, such as BIRC5, SURVIVIN, Bcl‐2, ABCG2, etc. Therefore, it is very likely that the regained sensitivity of CSCs to drug treatment observed both *in vitro* and *in vivo* due to Pin1 downregulation may be a collaborative result of impaired drug efflux and cell survival proteins, which are under the direct control of NOTCH1. G3 aggressive and poorly differentiated tumors are characterized by a large population of CSC overexpressing both Pin‐1 and NOTCH1 that may be responsible for drug resistance by simply safeguarding the self‐renewal and expansion of the CSC pool. A very recent study found that Pin‐1 not only plays a key role in regulating the stability of various phosphoproteins, including most FBXW7 substrates, but can also directly regulates the stability and/or function of FBXW7 or any other F‐box protein. Pin‐1 interacts with FBXW7 in a phosphorylation‐dependent manner and facilitates its degradation by preventing the dimerization of FBXW7. Pin‐1 overexpression in tumors reduces FBXW7 levels and suppresses the ability of FBXW7 to repress proliferation and oncogenic transformation. In contrast, Pin‐1 deficiency leads to FBXW7 overexpression that subsequently limits MCL‐1 expression, thereby sensitizing the tumor cells to drugs like Taxol. Although it is clear that Pin‐1 can mediate chemoresistance in tumors but it remains to be investigated whether Pin‐1 drives the process in CSCs only through the FBXW7/NOTCH1‐axis or by modulating other pathways as well.^55^, ^82^

Lung cancer is the most common type of malignancy worldwide and close to 80% of all lung cancers are categorized as NSCLC that remains the principal cause of lung cancer‐related mortalities. Several signaling pathways have been attributed to play a key role in NSCLC chemoresistance including NOTCH‐1, JAK/STAT, PI3K/Akt, Wnt, TGF‐β, etc. There exists a strong correlation between FBXW7, NOTCH, and miR‐223 in mediating NSCLC resistance against the drug erlotinib. FBXW7 is one of the principal targets of miR‐223 and is well known to play a key role in carcinogenesis by facilitating the degradation of various oncoproteins, which may be involved in MDR, such as c‐Myc, Cyclin‐E, NOTCH, c‐Jun, mTOR, MCL1, etc. Several studies have earlier highlighted the role of miR‐223 in regulating tumor cell sensitivity to chemotherapeutic drugs, although its precise role in promoting tumor resistance to the effects of epidermal growth factor receptor tyrosine kinase inhibitors (EGFR‐TKIs) used in the treatment of NSCLC remains undeciphered. To answer this question, a recent study investigated the pathological significance of miR‐223 both in the parental NSCLC cell line (HCC827) and erlotinib‐resistant HCC827 cell line (HCC827/ER). miR‐223 expression was found to be significantly upregulated in HCC827 cells when compared to HCC827/ER cells. Furthermore, siRNA‐mediated silencing of either Akt or NOTCH and miR‐223 expression results in decreased resistance of HCC827/ER cells to erlotinib. On the contrary, forced miR‐223 overexpression triggers cell resistance in HCC827 cells to erlotinib, suggesting a very crucial role of miR‐223 in modulating erlotinib response. Further investigation revealed that miR‐223 promotes erlotinib resistance in NSCLC cell lines, primarily by downregulating FBXW7 expression, which subsequently results in increased NOTCH activity in these cells. Moreover, FBXW7 overexpression reverses the observed phenotype and limits the erlotinib resistance in HCC827/ER cells. Collectively, these observations suggest that NSCLC cells upregulate miR‐223 to facilitate erlotinib resistance in NSCLC cell lines, primarily through the FBXW7/NOTCH pathway. Therefore, the miR‐223/FBXW7/NOTCH axis may outline a promising therapeutic target for NSCLC patients whose neoplasms are resistant to erlotinib. Further studies in patient‐derived tumor xenograft (PDX) animal models may be needed to reinforce that this mechanism is indeed responsible for acquired resistance to erlotinib *in vivo*.^83^

## 7 FBXW7 AS A DIAGNOSTIC MARKER IN TUMORS?

In simple terms, diagnostic markers are biochemical pointers to the presence or progress of a disease. However, in a clinical setting, it often refers to a protein that can be detected in plasma and body fluids. Tumor markers are measurable biomolecules that can be attributed to a malignancy and are either secreted directly by cancer cells (tumor‐derived) or by the body as a whole in response to a tumor (tumor‐associated). Tumor markers may not be used as the primary means of cancer diagnosis, rather they can be used as an assay to support the diagnosis. Cancer is a group of diseases requiring alterations in the homeostatic status and expression of a discreet array of genes that confer a survival advantage and undiminished proliferative capability to somatic or germinal cells. Primarily, alterations in three principal classes of genes, namely (proto) oncogenes, tumor suppressor genes, and DNA repair genes drive the formation of tumors, which escape the natural and inherent apoptotic mechanism(s). Although tumor diagnostic markers may be inconclusive as screening assays for the detection of occult (hidden) cancers, once a particular tumor has been located using a marker, the marker can be used as a means of observing the success (or failure) of treatment. Furthermore, the marker expression level may also reveal the extent (the stage) of the disease, indicating how quickly the cancer is likely to spread and thereby assist in framing treatment protocols.^8^ As discussed in great detail, FBXW7 is the substrate recognition component of an evolutionary conserved SCF‐type E3 ubiquitin ligase that brings about the degradation of several key proto‐oncogenes, such as c‐Myc, Cyclin E, NOTCH, and c‐Jun. Essentially, FBXW7 is itself a tumor suppressor, the regulatory network of which is disturbed in many human carcinomas. Diverse cancer‐associated mutations in FBXW7 and its substrates have been observed, and loss of FBXW7 function has been found to induce chromosomal instability during tumorigenesis. FBXW7 displays a unique mutational spectrum in tumors, and different types of mutations can drive substrate‐specific consequences including dominant‐negative effects.^84^ The question that we ask here is, can FBXW7 serve as a diagnostic marker in cancer, given its deregulated expression patterns in a range of tumors?

Several groups are mapping FBXW7 expression patterns *in vivo* in tumors and emerging evidences are encouraging enough to suggest that FBXW7 may be used as a diagnostic marker at least in some forms of tumor. For instance, there are very few prognostic markers, which can be relied upon to indicate the progression of gastrointestinal stromal tumors (GISTs). GISTs are the most common mesenchymal tumors of the gastrointestinal tract, which originate from the interstitial cells of Cajal lineage due to activating mutations affecting c‐Kit or platelet‐derived growth factor receptor alpha (PDGF). FBXW7 regulates tumor size and tumor cell mitosis and hence plays a key role in the progression of GISTs. In hepatocellular and colorectal carcinomas, FBXW7 under‐regulation affects c‐Myc and NOTCH1 expression levels, which can be directly correlated to cell proliferation, migration, invasion, and hence poor prognosis. Koga et al. reported that FBXW7 expression may be a key etiological factor driving recurrence‐free survival (RFS) in intermediate or high‐risk patients, when compared to expression levels of c‐Myc, phosphorylated c‐Myc, and NOTCH1 in some tumor subsets. And therefore, FBXW7 may be explored as a novel prognostic marker during GIST progression. However, there may be certain limitations: First, FBXW7 mutations are frequent in several cancer cells, while FBXW7 mutations associated with GISTs are still being elucidated. Second, the precise molecular mechanism of FBXW7 deregulation in GISTs remains unclear. Nevertheless, as observed through multivariate analysis, FBXW7 expression in GISTs may be investigated further as an independent predictive marker of RFS following surgery in patients who shall benefit from adjuvant therapy better than conventional risk stratification in a clinical environment.^85^ Unfortunately, there are limited studies that have investigated the prospect of using FBXW7 as diagnostic marker during GIST progression in a large patient cohort. A recent study evaluated the effectiveness of FBXW7 as a clinical marker in a cohort of gastric carcinoma (GC) patients on postoperative chemotherapy protocols. The study found that gastric tumors expressing low levels of FBXW7 were the ones responsible for metastatic progression and poor prognosis. Furthermore, immunohistochemistry (IHC) analysis found that FBXW7 overexpression could be strongly correlated to improved response to chemotherapy. This opened a new avenue for research where low FBXW7 expression patterns can be explored as a predictive tool for poor prognosis in patients with GC. Besides, given the range of chemotherapeutics reported in this large database study, FBXW7 was found to serve as an important predictor of chemotherapeutic responses. Nevertheless, more investigations might be warranted to testify the effectiveness of FBXW7 as a diagnostic marker during or post‐chemotherapeutic regime, not only in GIST but also during the progression of a wide range of cancers.^86^

High Tribbles homolog 2 (TRIB2) expressions can be associated in part with NOTCH1 and/or FBXW7 mutations in pediatric T‐ALL group, although there are evidence for other drivers in operation. TRIB protein family members encode pseudo‐kinase proteins that are highly conserved and function as adaptors in signaling activities for key cellular processes. Reports demonstrate that TRIB2 knockdown can limit tumor growth and proliferation and limit drug resistance in patients suffering from human chronic myelogenous leukemia (CML), mainly through the ERK/STAT3 pathway. Moreover, high TRIB2 expression correlates well with increased NOTCH1 activity in some adult and pediatric T‐ALL cases. Interestingly, high TRIB2 expression alone may adequately distinguish a T cell profile among all subtypes of leukemia and therefore, may act as potential marker for malignancies with T cell features. Lastly, the interactions between TRIB2 and other T cell signaling pathways may be useful in uniquely identifying leukemia subtypes and can further advance our understanding of T‐ALL pathology.^87^, ^88^

## 8 NOVEL THERAPEUTICS TARGETING DEREGULATED NOTCH‐FBXW7 INTERACTION IN TUMORS

Over the last decade, enormous progress has been in the field of cancer therapeutics in addition to the most common and conventional therapies that include surgery, radiation, and chemotherapy. There are drawbacks to these conventional therapeutic protocols, which cause a lot of physical as well as psychological stress among infirm. Chemotherapeutic drugs trigger different types of toxicity in the patient's body, including cardiotoxicity, hematotoxicity, neurotoxicity, gastrointestinal toxicity, hair follicle toxicity, nephrotoxicity, and many more. Moreover, these drugs target rapidly multiplying cells and are sometime unable to distinguish between normal cells and tumor cells, thereby limiting their maximum allowable doses. On the contrary, these chemotherapeutic agents get swiftly eliminated through the ADME mechanisms, and therefore, administration of a high dosage of the drug is needed to circumvent rapid elimination and for widespread distribution of the drug to the tumor area. Subsequent research advancements have led to the identification of various alternative modalities, such as immunotherapy, liposomal therapy, targeted therapy, hormone therapy, and stem cell therapy, to mitigate the detrimental effects of these drugs. The use of biological molecules to activate the immune system that is severely compromised in patients has been an area of intense deliberation. In contrast to chemotherapy, targeted use of biomolecules is highly efficient against tumor cells and come with limited side effects. Therefore, targeted use of biological and physiological therapies intending to reduce side effects and increasing the long‐lasting efficacy will be extremely beneficial in cancer treatment going forward.^89^, ^90^ Deregulated FBXW7 and NOTCH interactions can be targeted through novel biomolecules to improve prognosis in tumors. For instance, Chromosome Maintenance Region 1 (CRM1) inhibition that can lead to nuclear retention of tumor suppressor proteins has been explored as a therapeutic strategy in pancreatic ductal adenocarcinoma (PDAC). It was found that CRM1 inhibitors exert their therapeutic effects both *in vitro* and *in vivo* through a novel FBXW7 nuclear retention mediated NOTCH1 suppression. CRM1 also known as Exportin 1 (Xpo1) is a protein that has been found to be upregulated in PDAC cells, and blocks the activity of tumor suppressor protein (TSP) through constant nuclear export. Targeting CRM1 through specific inhibitors of nuclear export (SINE) has earlier shown promise in the inhibition of pancreatic cancer cell proliferation and growth. Most importantly, the inhibitor SINE KPT‐185 inhibits PDAC growth, migration, invasion, and triggers apoptosis and G2‐M cell cycle arrest at very low concentrations. The activity of KPT‐185 is strongly correlated to the nuclear retention of FBXW7; which degrades nuclear N1‐ICD, thereby leading to reduced expression of tumor‐promoting markers like Cyclin‐D1, c‐Myc, VEGF, and Hes‐1. The orally bioavailable form of SINE (KPT‐251) has shown tremendous antitumor activity in a Colo‐357 PDAC xenografts model.^91^ Likewise, there is a panel of bioactive compounds that have shown to target the deregulated F‐box protein in a range of tumors and are listed in Table 2 below.

**TABLE 2:** Summary of bioactive compounds mediated targeting of SCF type E3 ligase components in cancer

Compounds	F‐box protein	Associated mechanisms	Associated cancer	References
KL001	Fbxl3	Competes for binding in the FAD pocket of CRYs and prevents Fbxl3 binding	Cancer, cardiovascular and metabolic diseases	92, 93
SINE KPT‐185	FBXW7	Inhibits nuclear export of FBXW7, enhances nuclear retention of FBXW7 and degrades NOTCH1	Pancreatic cancer	91
Genistein	FBXW7	Inhibits miR‐223 expression and elevates its target FBXW7 expression	Leukemia and lymphoma	94
Oridonin	FBXW7	Increases FBXW7 expression, activates GSK3, and facilitates c‐Myc turnover	Leukemia and lymphoma	95
Erioflorin	κ‐TrCP	Inhibits the interaction between κ‐TrCP and tumor suppressor Pdcd4	Cancer, general	96
GS143	κ‐TrCP	Disrupts interaction between phospho‐IκBκκ and κ‐TrCP and suppress IκBκ ubiquitylation	Inflammation	97
SMIP004	Skp2	Downregulates Skp2	Prostate cancer	98
Compound A (CpdA)	Skp2	Interferes Skp2‐Skp1 interaction and stabilizes p27	Multiple myeloma	99
Compound #25	Skp2	Binds to Skp2 and prevents Skp2‐Skp1 interaction	Cancer, general	100
C1, C2, C16, C20	Skp2	Binds to a pocket formed by Skp2 and Cks1 to block substrate binding	Metastatic melanoma, prostate, breast, ovarian, and lung cancer	101
Curcumin	Skp2	Natural agents, inhibit Skp2 expression	Breast and prostate cancer	102, 103, 104
BC‐1258	Fbxl2	Inhibits binding between Fbxo3 and Fbxl2, stabilizes Fbxl2 and promotes Aurora B degradation	Inflammation	105
BC‐1215	Fbxl2	Inhibits the Fbxo3 and Fbxl2 binding	Inflammation	106
Quercetin	Skp2	Natural agents, inhibit Skp2 expression	Breast and prostate cancer	102, 103, 104
Lycopene	Skp2	Natural agents, inhibit Skp2 expression	Breast and prostate cancer	102‐104
Silibinin	Skp2	Natural agents, inhibit Skp2 expression	Breast and prostate cancer	102‐104
Vitamin D	Skp2	Natural agents, inhibit Skp2 expression	Breast and prostate cancer	102‐104
Epigallocatechin gallate (EGCG)	Skp2	Natural agents, inhibit Skp2 expression	Breast and prostate cancer	102‐104

## 9 CONCLUDING REMARKS

FBXW7 and NOTCH based interactions cater to a panel of cellular processes in the mammalian system, which under ideal conditions are essential for maintaining normal cellular homeostasis. However, FBXW7 and NOTCH activity can be deregulated due to incriminating factors like a mutation that disturbs the homeostatic state, thereby driving oncogenic transformation. In this current review, we discussed about the recent advancements made in studying the disease‐critical interactions involving FBXW7 and NOTCH, which aberrantly drive key biological processes, such as cell cycle, cellular proliferation, metastasis, apoptosis, etc. Although at the very early stage of research, we explored the possibility of utilizing FBXW7 as a novel diagnostic marker in tumors. The FBXW7‐NOTCH interactome is extremely complex but from a therapeutic perspective it holds tremendous promise. In that aspect, several biomolecules are now being investigated for their unique ability to selectively target proteins involved in the interactome. Moving forward, we sincerely believe that unraveling the intricacies of this complex interactome may provide a therapeutic outlook for patients suffering from both solid and nonsolid tumors and displaying extremely poor prognosis.

## AUTHOR CONTRIBUTIONS

**N. K. J.:** Conceptualization; data curation; writing‐original draft; writing‐review and editing. **R. K.:** Conceptualization; data curation; writing‐original draft; writing‐review and editing. **S. K. J.:** Conceptualization; data curation; writing‐original draft; writing‐review and editing. **S. O.:** Writing‐review and editing. **A. S.:** Writing‐review and editing. **V. S. R. R.:** Writing‐review and editing. **P. P.:** Writing‐review and editing. **D. K. C.:** Writing‐review and editing. **G. G.:** Writing‐review and editing. **S. K. S.:** Writing‐review and editing. **K. R. P.:** Writing‐review and editing. **P. H.:** Writing‐review and editing. **S. K. S.:** Writing‐review and editing. **J. R.:** Writing‐review and editing. **K. K. K.:** Writing‐review and editing. **K. D.:** Writing‐review and editing. **S. D.:** Writing‐review and editing.

## CONFLICT OF INTEREST

There is no conflict of interest.

## ETHICAL STATEMENT

Not applicable.

## Data Availability

Data sharing is not applicable to this article as no new data were created or analyzed in this study.
